# Stable, high-performance, dendrite-free, seawater-based aqueous batteries

**DOI:** 10.1038/s41467-020-20334-6

**Published:** 2021-01-11

**Authors:** Huajun Tian, Zhao Li, Guangxia Feng, Zhenzhong Yang, David Fox, Maoyu Wang, Hua Zhou, Lei Zhai, Akihiro Kushima, Yingge Du, Zhenxing Feng, Xiaonan Shan, Yang Yang

**Affiliations:** 1 NanoScience Technology Center, University of Central Florida, Orlando, FL 32826 USA; 2 Electrical and Computer Engineering Department, W306, Engineering Building 2, University of Houston, Houston, TX 77204 USA; 3 Physical and Computational Sciences Directorate, Pacific Northwest National Laboratory, Richland, WA 99352 USA; 4 Department of Chemistry, University of Central Florida, Orlando, FL 32826 USA; 5 School of Chemical, Biological, and Environmental Engineering, Oregon State University, Corvallis, OR 97331 USA; 6 X-ray Science Division, Argonne National Laboratory, Lemont, IL 60439 USA; 7 Department of Materials Science and Engineering, University of Central Florida, Orlando, FL 32826 USA; 8 Advanced Materials Processing and Analysis Center, University of Central Florida, Orlando, FL 32826 USA; 9 Energy Conversion and Propulsion Cluster, University of Central Florida, Orlando, FL 32826 USA

**Keywords:** Batteries, Batteries

## Abstract

Metal anode instability, including dendrite growth, metal corrosion, and hetero-ions interference, occurring at the electrolyte/electrode interface of aqueous batteries, are among the most critical issues hindering their widespread use in energy storage. Herein, a universal strategy is proposed to overcome the anode instability issues by rationally designing alloyed materials, using Zn-M alloys as model systems (M = Mn and other transition metals). An in-situ optical visualization coupled with finite element analysis is utilized to mimic actual electrochemical environments analogous to the actual aqueous batteries and analyze the complex electrochemical behaviors. The Zn-Mn alloy anodes achieved stability over thousands of cycles even under harsh electrochemical conditions, including testing in seawater-based aqueous electrolytes and using a high current density of 80 mA cm^−2^. The proposed design strategy and the in-situ visualization protocol for the observation of dendrite growth set up a new milestone in developing durable electrodes for aqueous batteries and beyond.

## Abstract (web summary)

Metal anode instability due to several intrinsic factors limits their widespread use in energy storage. Here, the authors report a 3D alloy anode via a universal alloy electrodeposition approach to overcome the anode instability issues and demonstrate a seawater-based aqueous battery.

## Introduction

The strong safety concerns caused by the decomposition of organic electrolytes are challenging non-aqueous lithium-ion battery (LIB) communities, posing formidable barriers to reliable electric vehicles (EVs) and personal electronics^1^. Alternatively, emerging metal-anode-based aqueous batteries are attracting increasing attention due to the high-safety of nonflammable electrolytes^2–4^ and environmental benignity^5–7^. More importantly, when coupled with earth-abundant elements (e.g., O_2_ and S) at the cathodes, high-energy-density are possible, leading to cutting-edge technology for the advanced battery systems that exceed the energy density of 500 Wh kg^−1^ required for the future EVs^8^. However, inhomogeneous metal plating and electrochemical instability at the liquid-solid (electrolyte/metal anode) interface severely jeopardize the performance and life span of aqueous batteries^9–13^. The inhomogeneous metal plating incurs uncontrollable dendrite growth on the anode surface during charge/discharge cycling, inevitably leading to low Coulombic efficiency (CE), poor cyclability, and even operating failure caused by short-circuit^14–16^. In recent years, various strategies have been suggested to resolve the aforementioned interfacial instability issues of metal anodes in aqueous batteries from the perspectives of materials science and surface chemistry, including structural optimization^17^, surface modification^18^, artificial solid-electrolyte interphases (SEI)^19^, understanding the metal-based battery chemistry, and controlling metal plating^15,20^. Nevertheless, progress in stabilizing metal anodes is still in early infancy, which encourages the aqueous battery communities to explore more efficient and universal strategies for addressing the issues of inhomogeneous metal plating and interfacial instability.

On the other hand, from the perspective of electrolyte chemistry, the solvents and salts used in aqueous electrolytes are among the most important components in aqueous batteries that determine their performance^21^. In practice, deionized (DI) water and high-purity water are commonly used solvents^16,21^ in aqueous batteries to achieve well-controlled battery chemistry by eliminating the interference of hetero-ions (e.g., Ca^2+^, Mg^2+^, Na^+^, SO_4_^−^, Cl^−^, NO_3_^−^, F^−^, etc.) on the battery stability^21^. Besides, blended salts have been used in the electrolytes to improve the electrochemical performance of aqueous batteries^22,23^ by tuning the composition of cations and anions in the electrolyte, thereby achieving high ionic conductivity^22,24,25^. However, the complexity of the electrolyte components used in those strategies makes them economically less competitive than current rechargeable battery technologies for industrial-level applications.

Herein, a three-dimensional (3D) alloy anode has been proposed and demonstrated to resolve the interfacial instability issues and improve the electrochemical performance of aqueous batteries using low-cost seawater-based electrolytes. Different from the strategies using surface passivation layers to prevent dendrite growth in non-aqueous lithium electrochemical systems^26,27^, we propose a strategy that will efficiently minimize and suppress the dendrite formation in aqueous systems by controlling: (1) the surface reaction thermodynamics with the favorable diffusion channel of Zn on the Zn_3_Mn alloy, and (2) the reaction kinetics through the 3D nanostructures on the electrodes, at the same time. The relatively higher binding energy on the surface of the Zn_3_Mn alloy could help to guide and regulate Zn nucleation and growth and minimize the dendrite formation at the early stage of the deposition. The porous 3D nanostructure will be favorable for controlling the Zn^2+^ ions diffusion kinetics, further minimizing the dendrite growth throughout the entire deposition process. We designed an optical in-situ visualization protocol that could exactly mimic the actual electrochemical conditions in the aqueous systems. Using this protocol, we observed reversible metal plating and stripping processes within the 3D Zn-Mn anode under different aqueous electrolytes including seawater. Also, theoretical (density functional theory, DFT) and experimental (microscopic and spectroscopic) studies proved that the proposed 3D alloy anode has outstanding interfacial stability achieved by the favorable diffusion channel of Zn on the alloy surface. As a proof-of-concept, the proposed Zn-Mn alloy anodes were demonstrated to be ultra-stable during the Zn plating and stripping processes, leading to durable and dendrite-free electrodes for aqueous battery even under a high current density of 80 mA cm^−2^. This work presents a big step towards high-performance, high-flexibility, and reliable rechargeable batteries using seawater-based electrolytes. This work also provides a further understanding of aqueous battery chemistry that will advance the use of aqueous batteries in the renewable energy field and beyond.

## Results

### Preparation and characterizations of alloy anode

**Fig. 1:**
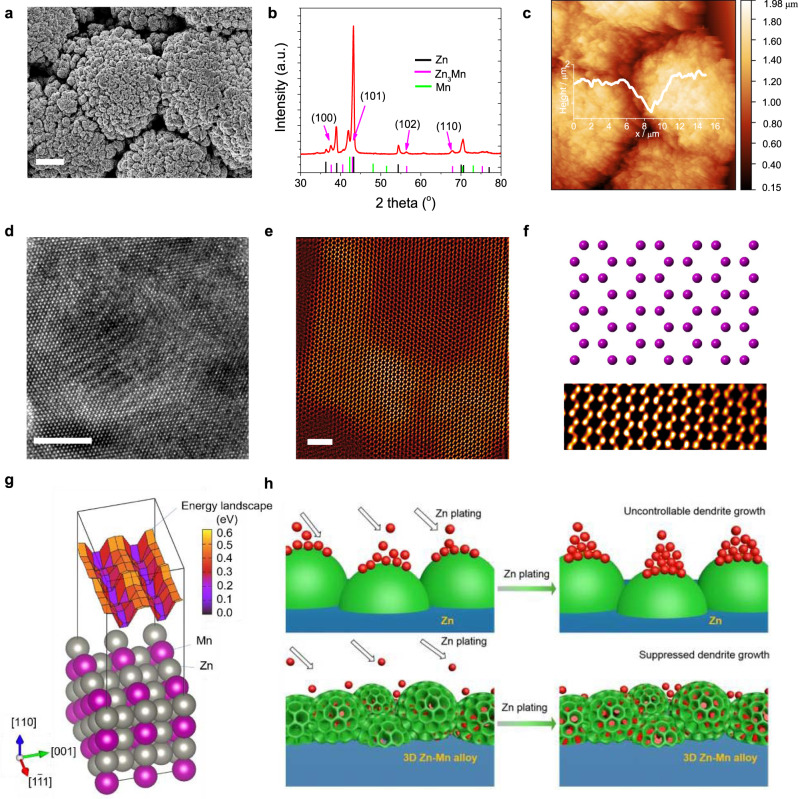
Preparation and characterizations of the Zn-Mn alloy anode. **a** SEM image. The scale bar: 10 μm. **b** XRD pattern. **c** AFM image. **d** HRTEM image of Zn_3_Mn viewed along [001] direction. The scale bar: 10 nm. **e**, **f** HAADF-STEM image and the corresponding atomic crystal structure. The scale bars: 2 nm. The purple balls in the crystal structure model represent the co-occupied Zn/Mn atoms. **g** Atomic structure and the surface ad-atom energy landscape of Zn_3_Mn. **h** Schematic illustration of Zn plating processes on Zn anode (top) and Zn-Mn anode (bottom).

An alloy electrodeposition approach was developed to prepare 3D structured Zn-Mn anodes as proposed in this work. This method can be used as a universal strategy for synthesizing various alloy anodes by adjusting the composition of deposition solution, applied deposition current or voltage, and deposition time. In this work, we focused on validating the proposed concept of 3D alloy anode by studying the electrochemical performance of Zn-Mn anode. Compared with Zn^2+^/Zn, the standard equilibrium potential of Mn^2+^/Mn is much lower (Supplementary Table 1), enabling the Zn deposition on the surface of Zn-Mn alloy unfavorable for Zn dendrite formation due to the electrostatic shield effect^10,28^. We also demonstrated the potential extension of this alloy electrodeposition strategy by showcasing another anode − Zn-Cu alloy at the end of this paper. We further suggested other alloys beyond Zn-Mn and Zn-Cu, such as Zn-Ni, Zn-Co, Zn-Fe, Zn-Mg, etc., based on their high corrosion resistance among the typical Zn-based alloys^29^, which will inspire more follow-up works from the battery and materials science communities. The electrodeposition of 3D Zn-Mn alloy was performed in a two-electrode electrochemical cell by a galvanostatic method (more experimental details in the Methods section). Continuous hydrogen (H_2_) bubbles were observed during the alloy electrodeposition because of water dissociation incurred by the extremely high current density of 0.3 A cm^−2^ used in this work. We varied the electrodeposition time from 10 min to 40 min and found that the evolved H_2_ bubbles served as gaseous templates for the 3D structure formation following the Stranski-Krastanov mechanism (Supplementary Fig. 1 and Supplementary Discussion 1)^30^. The morphologies of the Zn-Mn alloy changed from an isolated island-like structure to an interconnected 3D structure with a cauliflower-like surface (Supplementary Fig. 2). Based on the microscopic characterizations, the proposed alloy electrodeposition processes mainly include: (i) co-electrodeposition of various ions (Zn^2+^ and Mn^2+^);^31^ (ii) H_2_ bubbles evolution at the solid-liquid interface leading to the formation of the 3D structure (Fig. 1a and Supplementary Fig. 3). Meanwhile, the hierarchical pores on the surfaces of the cauliflower-like 3D structures (Supplementary Fig. 4) are beneficial for the facilitated mass transfer during charge/discharge cycling^32,33^. XRD pattern (Fig. 1b) and energy-dispersive X-ray spectroscopy (EDS, Supplementary Fig. 5) elemental mapping confirm the formation of Zn-Mn alloy. The main peaks in the XRD pattern primarily correspond to the phase of P63/mmc(194)-hexagonal Zn_3_Mn (note: in the following discussion Zn-Mn alloy and Zn_3_Mn denote the same material). The topography of the Zn-Mn alloy was observed with atomic force microscopy (AFM, Fig. 1c and Supplementary Fig. 6) over a 20 × 20 μm area. The cauliflower-like 3D structures show a hierarchical roughness due to the co-existence of both micro- and nanoscale pores on the surface (Supplementary Fig. 7). After Zn plating, the hierarchical roughness does not show a significant change with the root mean squared (RMS) of 25 nm and 32 nm calculated for the Zn-Mn anodes before and after Zn plating, respectively. In contrast, the AFM topographies of the pristine Zn after Zn plating indicate that the dendrites formation would occur easily even in a range of area capacities from 1.0 mAh cm^−2^ to 5 mAh cm^−2^ (Supplementary Fig. 8). The AFM topographies prove that the inhomogeneous dendrite growth is suppressed in the 3D Zn-Mn alloy by the Zn plating primarily into the hierarchical pores. High-resolution transmission electron microscopy (HRTEM, Fig. 1d) of Zn_3_Mn shows a well-crystallized alloy structure. In addition, atomic resolution high angle annular dark-field (HAADF) scanning transmission electron microscopy (STEM) images (Fig. 1d–f) show the unique structure of Zn_3_Mn, which provides the fast diffusion path for the cations.

Hydrophilic surfaces on metal anodes are essential for homogeneous Zn plating in aqueous electrolytes^34^. The surface wettability of Zn_3_Mn and Zn anodes with different solvents (DI water and seawater) was characterized by the contact angle (CA) goniometry (Supplementary Figs. 9–11). It is noteworthy that the 3D Zn_3_Mn anodes with unique 3D structure and nature of alloy possess superhydrophilic characteristics (CA: 0°; Supplementary Discussion 2) compared with the pristine Zn anode (CA: 103 ± 0.5°), enabling the considerably enlarged electrode/electrolyte contact, facilitating the mass transfer, and thus achieving homogeneous Zn plating. After the calendering process, the surface structure and morphology of the Zn-Mn alloy electrode remain barely changed even under high pressure of 80 MPa, indicating excellent mechanical stability (Supplementary Fig. 12). Even under much higher pressures of 160 MPa and 200 MPa, only the top-surface structure of Zn-Mn alloy was squeezed. The basic shape of the 3D structured Zn-Mn alloy with a large number of voids and trenches remains stable, which provides free space for depositing Zn metal. Furthermore, density functional theory (DFT) calculations were employed to understand the role of the alloy phase in regulating Zn nucleation and growth in the plating process. The calculated binding energy of a Zn atom on the surface of Zn_3_Mn is 1.42 eV, a higher value than the Zn surface (1.12 eV), indicating that the Zn_3_Mn phase could be an ideal matrix to guide the Zn plating because of a stronger interaction between Zn and Zn_3_Mn. In contrast, the pristine Zn shows a weaker interaction with Zn atoms resulting in a greater tendency for dendrite growth. The theoretical understanding of Zn diffusion on the Zn_3_Mn surface is demonstrated in Fig. 1g, showing the surface structure of Zn_3_Mn and the Zn-ad-atom energy landscape. The energy landscape clearly shows two channels with lower energy and small ripples separating the local minima located at the surface lattice points. The activation barrier for Zn diffusion is 0.24 ± 0.025 eV, comparable to that of Li diffusion inside graphite and graphene^35–37^, indicating that Zn_3_Mn could be a promising host for Zn diffusion. Particularly, the fast Zn diffusion channel inside the Zn-Mn alloy with stronger binding contributes to a homogeneous Zn coverage on the electrode surface and therefore suppresses dendrite growth (Fig. 1h). In contrast, the Zn plating/stripping behaviors on the surface of the Zn anode are inhomogeneous, and subsequently favor the dendrite growth, also known as the “tip effect”^16^.

### Alloy anode stability under harsh electrochemical environments

**Fig. 2:**
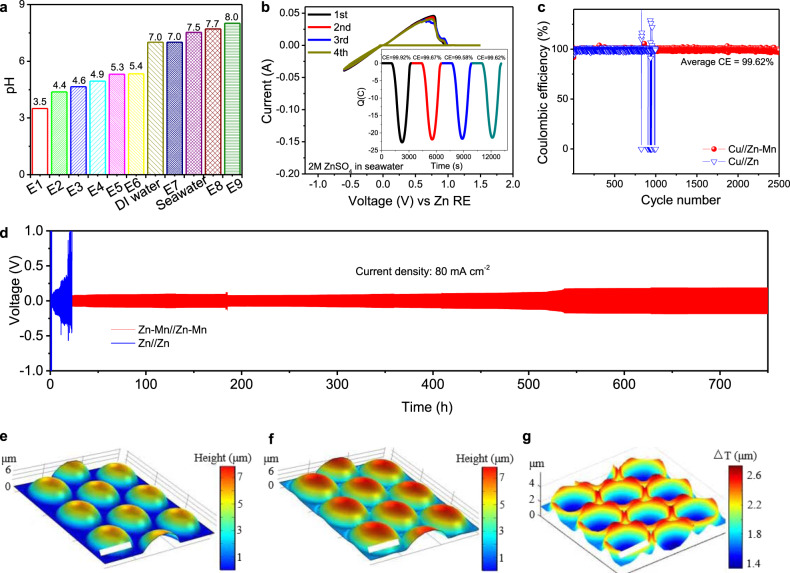
Electrochemical performance and Zn plating/stripping behaviors of Zn-Mn alloy in the aqueous electrolytes. **a** pH values of different electrolytes prepared using DI water and seawater as solvents. **b** Cyclic voltammetry curves of seawater-based electrolytes. Scan rate: 1 mV s^−1^. Working electrode: Pt. Reference and counter electrodes: Zn-Mn alloy. **c** Long-term galvanostatic cycling performance of Cu//Zn and Cu//Zn-Mn cells at a current density of 10 mA cm^−2^. **d** Long-term galvanostatic cycling performance of symmetric Zn-Mn and pristine Zn cells at a current density of 80 mA cm^−2^ (areal capacity: 16 mAh cm^−2^; Electrolyte: 2 M ZnSO_4_ in seawater). 3D COMSOL simulation: **e** Morphology of 3D Zn-Mn alloy in 3D COMSOL model before Zn plating. **f** Morphology of 3D Zn-Mn alloy after 50 s of Zn plating. **g** Thickness change after 50 s of Zn plating on the Zn-Mn alloy surface. Scale bars in **e**–**g**: 20 µm.

Traditional metal anodes used in aqueous batteries have poor stability under harsh conditions because of the accelerated corrosion, hetero-ions interference, and unexpected side-reactions. To further examine the electrochemical stability of Zn_3_Mn anode under harsh environments, seawater-based electrolytes consisting of complex compositions (3.5% saline water containing Na^+^, Mg^2+^, Ca^2+^, SO_4_^−^, Cl^−^, etc.) were adopted in this work. Another benefit of using seawater-based electrolyte is attributed to its earth abundance and almost free of charge (Supplementary Table 2)^38,39^, providing gigantic economic interest and competitiveness in the increasing energy storage markets. To systematically compare seawater-based electrolytes with conventional DI water-based electrolytes, we prepared nine kinds of aqueous electrolytes using DI water and seawater (Supplementary Fig. 13) as solvents for different metal salts (ZnSO_4_, MgSO_4_, NaSO_4,_ and MnSO_4_). In general, seawater-based electrolytes have higher pH levels than DI water-based electrolytes (Fig. 2a), making seawater a viable solvent for the naturally mild aqueous electrolytes. We used a three-electrode electrochemical cell with Pt as the working electrode and Zn_3_Mn alloy as the counter and reference electrodes to test the reversibility of Zn plating/stripping behaviors and electrochemical window in an electrolyte composed of 2 M ZnSO_4_ in seawater (Fig. 2b). The chronocoulometry curves show that the Zn plating/stripping is highly reversible with a nearly 100% CE (initial CE: 99.92%). A stable and wide electrochemical window up to 2.6 V was achieved by using a Zn_3_Mn anode in the seawater-based electrolyte without any electrolyte decomposition (Supplementary Fig. 14). The electrochemical stability window of aqueous electrolytes was explored by testing water dissociation potentials (Supplementary Fig. 15a), e.g., hydrogen evolution reaction (HER) and oxygen evolution reaction (OER), in a three-electrode system. The seawater-based electrolyte (2 M ZnSO_4_ in seawater) has a wider electrochemical window increased from 2.4 V to 2.6 V as compared with DI water-based electrolyte (2 M ZnSO_4_ in DI water). When using seawater as a solvent, the content of free water molecules decreases, which has been proven to be an effective strategy to expand the electrochemical stability window^21,40^. Moreover, the Zn_3_Mn electrode shows a significantly improved anti-corrosion ability in the seawater-based electrolyte as compared to the Zn electrode (Supplementary Fig. 15b), due to the synergistic effects as reported in the previous reports^41^. On the contrary, a vigorous electrolyte decomposition and much narrower electrochemical windows were detected by using pristine Zn anode in both DI water-based and seawater-based electrolytes (Supplementary Fig. 16). The CE of Zn plating/stripping processes was further evaluated via Cu//Zn (or Cu//Zn-Mn) cells using different aqueous electrolytes. A higher and more stable CE for Cu//Zn-Mn cells using different aqueous electrolytes was obtained (Supplementary Figs. 17 and 18). For the cycling performance of CE, the Zn-Mn alloy appears to have an average CE above 99.6% over 2500 cycles at a current density of 10 mA cm^−2^ (Fig. 2c), demonstrating the long-term durability of Zn_3_Mn anode in the seawater-based electrolyte. Furthermore, electrochemical impedance spectra (EIS) of Zn//Zn and Zn-Mn//Zn-Mn symmetric cells were examined to understand the charge transfer kinetics in different electrolytes. In seawater-based electrolyte, a remarkably reduced charge transfer resistance was achieved with a Zn-Mn//Zn-Mn symmetric cell (Supplementary Fig. 19a), which was much lower than that of Zn//Zn symmetric cell, indicating the facilitated reaction kinetics of Zn-Mn alloy. Similarly, the improved reaction kinetics was observed in the Zn-Mn alloy symmetric cells using DI water-based electrolytes (2 M ZnSO_4_ in DI water, Supplementary Fig. 19b) compared with pristine Zn. The nucleation and plateau overpotentials indicate the formation and growth thermodynamics of critical Zn atoms/clusters in the plating process^42,43^. The nucleation and plateau overpotentials (27 mV and 19 mV, respectively) for the Zn-Mn alloy are much lower than those of pristine Zn anode (47 mV and 30 mV, respectively), further confirming the regulated Zn plating dynamics for Zn-Mn alloy anode (Supplementary Fig. 19c). Moreover, the outstanding stability of Zn-Mn anode was further proved by galvanostatic cycling in the symmetric Zn-Mn//Zn-Mn cell under an extremely high current density of 80 mA cm^−2^, showing ultra-stable plating/stripping behaviors for over 1900 cycles. Whereas, the short-circuit of the symmetric Zn//Zn cell was observed only after 80 cycles within <30 h (Fig. 2d and Supplementary Fig. 20). The achieved great improvements in the electrochemical stability of metal anode under harsh environments validate our concept of using a Zn-Mn alloy for durable aqueous batteries. To further confirm the significance of Zn_3_Mn in the stabilized electrochemical performance, we prepared a 3D Zn@Zn anode (Zn foil coated with 3D Zn particles, Supplementary Fig. 21) as a control sample for electrochemical tests. The Zn plating/stripping profiles and cycling performance of symmetric 3D Zn@Zn cell (Supplementary Fig. 22) exhibit a large overpotential and failure caused by the dendrite growth and the corresponding internal short-circuit within <100 cycles at a low current density of 5 mA cm^−2^ and <250 h at a high current density of 80 mA cm^−2^. Ex-situ SEM observations (Supplementary Fig. 23) were performed to diagnose the Zn plating processes under different current densities from 1 mA cm^−2^ to 80 mA cm^−2^. The dendrites were observed from the surface of pristine Zn anode, while a smooth surface without dendrite growth was achieved on the 3D Zn-Mn alloy anode even under harsh conditions such as high current densities up to 80 mA cm^−2^. The demonstrated homogenous Zn plating double confirmed the favorable binding energy of Zn atoms and the fast Zn diffusion channel in the Zn-Mn alloy as suggested by the DFT calculations.

### In-situ visualization of Zn plating/stripping processes

**Fig. 3:**
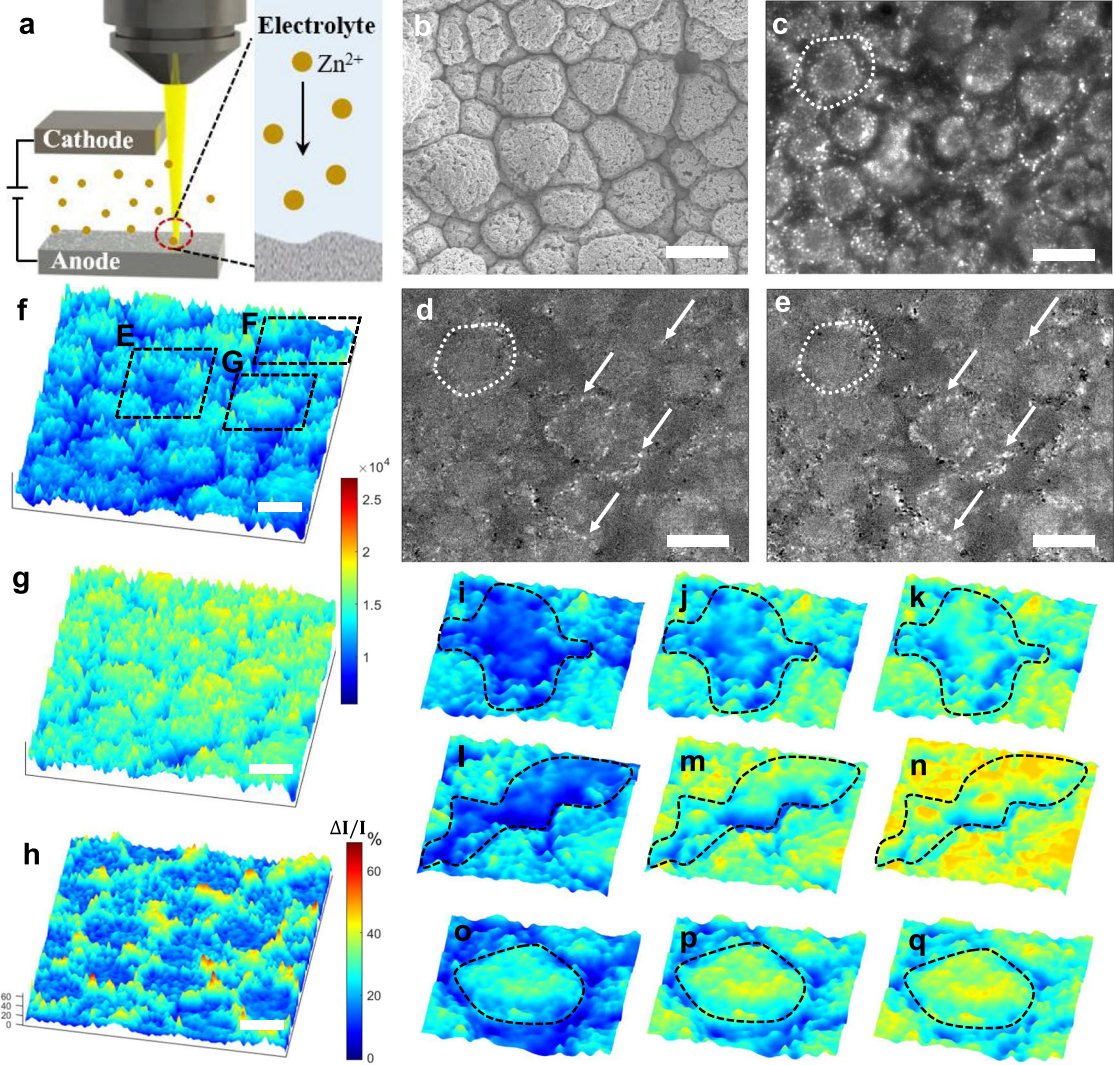
Zn plating dynamics on 3D Zn-Mn alloy imaged by in-situ optical microscope. **a** Schematic illustration of the experimental setup. **b** SEM image of 3D Zn-Mn alloy. **c**–**e** The early stage of Zn plating. Images were taken with a ×20 water immersion objective at 25 frames per second, and the experiment was performed at a current density of 80 mA cm^−2^. **c** shows the 3D Zn-Mn alloy before the experiment. **d**, **e** show the differential images at 10 s and 30 s, respectively, after the start of the experiment. **f**–**h** Zn plating on 3D Zn-Mn alloy. The experiment was performed at a current density of 80 mA cm^−2^ for 320 s. **f**, **g** are the images of 3D Zn-Mn alloy before and after Zn plating. **h** was calculated by (**g**–**f**)/**f** = (*∆I*/*I*). **i**–**q** Evolution of Zn plating on the 3D Zn**-**Mn alloy. **i**–**q** are from the three different regions of interest labeled in **f**, where **i**–**k**, **l**–**n**, and **o**–**q** correspond to regions **E**, **F**, and **G** in **f**, respectively. The images were taken at 0 s (**i**, **l**, **o**), 160 s (**j**, **m**, **p**), and 320 s (**k**, **n**, **q**). The black dashed lines in (**i**–**q**) circle out the trench regions (**i**–**n**) and the protruding regions (**o**–**q**). Scale bars: 10 μm.

The Zn plating and stripping processes in the aqueous batteries were in-situ visualized via a specially designed optical protocol (Fig. 3a). We imaged and compared the Zn plating and stripping processes on Zn-Mn alloy and pristine Zn electrodes under different current densities of 5–80 mA cm^−2^ in DI water and seawater-based aqueous electrolytes. Since the proposed Zn batteries were operated in 2 M ZnSO_4_ salt-based aqueous electrolytes, the refractive index mismatch between the air and electrolyte could cause severe distortion and blur the image due to the optical aberration. To minimize this effect, a ×20 water immersion objective was employed. We also constructed 2D and 3D finite element analysis models of electrochemical cells using COMSOL multiphysics (see Supplementary Discussion 3–6) and simulated the plating profile and thickness change on the Zn-Mn alloy. Concretely, Fig. 2e–g, Supplementary Fig. 24, and Supplementary Movie 1 illustrate the Zn plating process over the entire electrode surface at a current density of 80 mA cm^−2^. The results clearly show that initially, the trenches between the Zn-Mn particles filled up rapidly compared to the protruding areas in the early stage. After the initial stage, as the plating proceeded the surface became smoother and the deposition rate in the trenches decreased and resembled deposition in other regions (Supplementary Fig. 25). This process resulted in a uniform electrode surface, which was consistent with the ex-situ SEM characterizations (Supplementary Fig. 23).

For the unique in-situ visualization (Fig. 3a), the objective, top, and bottom electrodes were all immersed in the electrolyte. Since the visible light cannot penetrate through the thick Zn electrodes, we extended the bottom substrate and imaged the morphology changes by the reflected light intensity on the extended area. Note that the observation area did not directly face the top electrode, which could decrease the electric field and the current density in the area and cause the non-uniform electric field distribution. To minimize this effect, the observation region was chosen as close as possible to the projection of the top electrode. The distance from the edge of the top electrode’s projection to the center of the observation area was 200–400 μm which was significantly smaller than the separation of the electrodes (~2 mm), and the current densities in the observation area were similar to those inside the area of the electrodes. Comparing with other imaging techniques that have been utilized to study the metal deposition process, our in-situ optical system provide the direct top view that will clearly illustrate the deposition dynamics at different locations and is easy to use comparing with X-ray imaging which requires the synchrotron beamline^44^. To verify our assumption, a COMSOL model was developed to simulate the current density distribution across the bottom electrode (Supplementary Fig. 26). The current density at the observation area was at 95% of the maximum values between two electrodes, and this result further verified that the observation area can reflect the changes inside the battery electrodes.

The corresponding SEM and optical images of the 3D Zn-Mn alloy are shown in Fig. 3b, c, respectively. The optical image clearly shows the 3D structures of the Zn-Mn alloy with hierarchical pores on the surface. The contrast of the optical image originated from the morphologies and variations in reflectance at different locations. The brighter areas represent material protruding from the surface and high reflectivity, and the darker areas correspond to the trenches and low reflectivity. The shapes and sizes of the 3D structures match very well with those in the SEM images, demonstrating the feasibility of using optical microscopy to study the dynamic process of electrode reactions. To understand the Zn plating process on the 3D Zn-Mn alloy, a constant current was applied through the electrodes while the optical images were obtained at a certain framerate. The experimental conditions used in the in-situ optical microscopy study were identical to those in the Zn battery test. The entire Zn plating process was recorded, and the optical signal reflected the morphological changes during the charging and discharging processes. The spatial resolution in the optical imaging system could not resolve the initial nucleation sites that are smaller than the diffraction limit, however, it allows us to image and measure the entire Zn deposition process. This information provides critical evidence that we have utilized the morphology of 3D Zn-Mn alloy to control the reaction kinetics and minimize the dendrite formation.

The pristine Zn anode was studied first using the in-situ optical microscope (Supplementary Fig. 27a). The Zn dendrites nucleated on the electrode surface after 60 s of plating and continued to grow through the entire process (Supplementary Fig. 27b, c and Supplementary Movie 2). The results showed that the Zn plating on the pristine Zn surface was inhomogeneous, leading to both vertical and lateral growth of dendrites at a certain location (bottom right in Supplementary Fig. 27). To completely understand why the dendrite growth can be suppressed on the surface of this Zn-Mn alloy, we carefully studied the Zn plating dynamics using the in-situ optical microscopy and COMSOL simulation. In addition to the outstanding interfacial stability achieved by the favorable diffusion channel of Zn on the alloy surface, we found two additional reasons that are responsible for the suppressed dendrite growth.

First, in the early stage of deposition, the nano-voids embedded in the 3D Zn-Mn alloy structure helped to control the nucleation sites, leading to the random distribution of nucleation sites. Such a structure allows Zn to deposit easily inside the nano-voids. We found that Zn plating on the 3D Zn-Mn alloy showed completely different dynamics. In the early stage of Zn plating on the 3D Zn-Mn alloy, the Zn was mostly deposited inside the 3D structure with hierarchical pores (white dashed regions in Fig. 3c). Figure 3d, e and Supplementary Movie 3 show the differential optical images after 10 s (Fig. 3d) and 30 s (Fig. 3e) of Zn plating at a current density of 80 mA cm^−2^ on the 3D Zn-Mn alloy. Besides, bright spots corresponding to big nucleation sites (marked by white arrows in Fig. 3c) were observed in the trenches of the 3D structure. These phenomena were caused by the enhanced electric field and the high current density in the nano-voids of the 3D Zn-Mn alloy. To verify this hypothesis, a 2D COMSOL model was established to simulate the plating rate and the current density inside and around the nano-void (Supplementary Fig. 28), and the results showed that the Zn plating rate inside the nanostructure was much faster than that outside.

Secondly, the trenches in the 3D Zn-Mn alloy structures grew faster initially and formed a uniform electrode surface after plating. After the initial nucleation process, Zn started to deposit over the entire surface. However, the deposition rate varied with location: Zn deposited much faster in the trench compared with the deposition on the original structures (i.e., protrusions). Figure 3f shows the initial profile of 3D Zn-Mn alloy before Zn plating. After a long deposition time (320 s under a current density of 80 mA cm^−2^), the surface became smoother (Fig. 3g and Supplementary Movie 4). To further illustrate this effect, we chose three regions of interest (regions E, F, and G in Fig. 3f), and plotted their changes over time (Fig. 3i–q). The regions marked by the dashed black lines in Fig. 3i–n indicate the trenches on the electrode. These images at different time points (Fig. 3i–n) clearly show that the trenches were filled up quickly, and the final surface became much smoother. Furthermore, protruding regions (original structures), circled by the black dashed line in Fig. 3o–q also grew during the Zn plating process at a relatively slower rate. The percent intensity change over the entire surface is demonstrated in Fig. 3h. The changes in the trench were much bigger (40–60%) than that of the original structures (20%). This phenomenon was due to the uneven distribution of the electric field and the current density of the 3D alloy structure. Note that the color map represents the image intensity, and the bigger intensity corresponds to the higher structure altitude. We have also obtained 3D morphology using our in-situ optical microscope by taking pictures at a different focus plane of entire 3D structures and reconstruct the 3D morphology (Supplementary Fig. 29). The results further verify our conclusion that the deposition in the trench will be much faster than that on the protruding region which minimized the dendrite formation. Supplementary Movie 5–8 illustrate the Zn plating and the corresponding stripping processes on the same electrode (see Supplementary Discussion 7). We have also quantified the amount of Zn deposited onto the 3D Zn-Mn alloy electrode with no obvious dendrites formation. Supplementary Movie 9–13 shows that the Zn can be continuously deposited onto the substrate for >8200 s with 80 mA cm^−2^ without dendrites, and further demonstrates the superior performance of 3D Zn-Mn alloy substrate.

COMSOL models in 2D (Supplementary Fig. 30 and Supplementary Movie 14) and 3D were built to further understand the Zn plating processes over the 3D Zn-Mn structure as mentioned above. The half-spheres were used to mimic the 3D Zn-Mn alloy structure (Fig. 2e and Supplementary Fig. 24). The trenches on the 3D Zn-Mn alloy were filled in much faster than the protrude regions (Fig. 2e, f) and became smooth during the plating process. The deposition thickness changed much faster in the trenches (Fig. 2g) which perfectly reproduced the experimental results (Fig. 2e–g vs Fig. 3f–h). During the stripping process, the deposited Zn was removed and the original 3D surface almost completely recovered (Supplementary Fig. 31). This observation directly proves the absolute reversibility of the plating/stripping process by using Zn-Mn alloy, which has never been achieved by other metal anodes. Furthermore, in-situ optical microscopy was used to study the Zn plating in other aqueous electrolytes to further prove the stability of Zn-Mn alloy: (1) 2 M ZnSO_4_ in seawater; (2) 2 M ZnSO_4_ and 0.1 M MnSO_4_ in seawater; and (3) 2 M ZnSO_4_ and 0.1 M MnSO_4_ in DI water. No obvious difference was observed (Supplementary Movie 15–17 for electrolyte (1), (2), and (3), respectively), confirming the dendrite-free and ultra-stable nature of 3D Zn-Mn alloy anode for aqueous batteries. Pristine Zn was also tested with seawater-based electrolyte (electrolyte 2) to compare with the 3D Zn-Mn alloy anode (Supplementary Movie 18 and 19). The movies show that the pristine Zn has a much faster dendrite formation rate. In addition, Zn deposited unevenly leading to quickly formed dendrites on the electrode surface, which further demonstrated the advantages of 3D Zn-Mn alloy over pristine Zn metal anode for aqueous batteries.

### Dendrite suppression strategy: simultaneous control of thermodynamics and reaction kinetics for Zn plating

In recent years, the study of cathodes for aqueous Zn-air and Zn-ion batteries (ZABs and ZIBs) has been at the forefront of aqueous battery research^45–47^. Different strategies have been suggested and demonstrated to improve the interfacial stabilities. However, the critical issues of Zn metal anodes, such as dendrite growth, surface passivation and corrosion, etc., have been insufficiently addressed and continue to significantly challenge the development of high-performance and fully-rechargeable aqueous Zn batteries^48^.

In this paper, we have proposed and demonstrated a strategy that will efficiently minimize and suppress the dendrite formation by controlling: (1) the surface reaction thermodynamics with the favorable diffusion channel of Zn on the Zn_3_Mn alloy, and (2) the reaction kinetics through the 3D nanostructures on the electrodes, at the same time. The relatively higher binding energy on the surface of Zn_3_Mn alloy indicates that the alloy phase is an ideal matrix to guide and regulate Zn nucleation and growth and minimize the dendrite formation at the early stage of the deposition. On the other hand, the porous 3D nanostructure will help to control the Zn^2+^ ions diffusion kinetics, and further minimize the dendrite formation throughout the entire deposition process. The combination of both Zn_3_Mn alloy and 3D nanostructure provides the 3D Zn-Mn alloy electrode the superior performance on dendrite suppression and corrosion prevention.

A series of experiments have been conducted to demonstrate that the high-performance dendrite-free 3D Zn-Mn alloy is the result of both (1) Zn_3_Mn alloy which will control the surface reaction thermodynamics; and (2) the 3D nanostructure will control the 3D reaction kinetics. We have fabricated the flat Zn_3_Mn electrode by mechanically pressing the 3D Zn-Mn alloy and imaged the Zn deposition with our in-situ optical microscope. The results show that the Zn deposition happens on the flat Zn-Mn alloy area immediately and there is no obvious dendrite formed within 900 s at 80 mA cm^−2^ (Supplementary Fig. 32 and Supplementary Movie 20). Comparing with the pristine Zn electrode, which starts to show dendrite formation after just 100 s (Supplementary Fig. 27 and Supplementary Movie 2), the flat Zn-Mn alloy shows the good capability to control the surface reaction and suppress the dendrite formation. On the other hand, the 3D Zn-Mn alloy does not show obvious dendrite formation after 8200 s of Zn deposition (Supplementary Fig. 33 and Supplementary Movie 9–13) under the same experimental conditions. Besides, we have fabricated a 3D Zn substrate without Mn, and the result shows improved performance comparing with the pristine Zn surface but still outperformed by the 3D Zn-Mn alloy (Supplementary Fig. 22). These results indicate that by coupling with the Zn-Mn alloy composition, the 3D nanostructures help to control the deposition kinetics and further minimized the dendrite growth.

### Electrochemical performance of Zn-Mn anode in aqueous Zn batteries

**Fig. 4:**
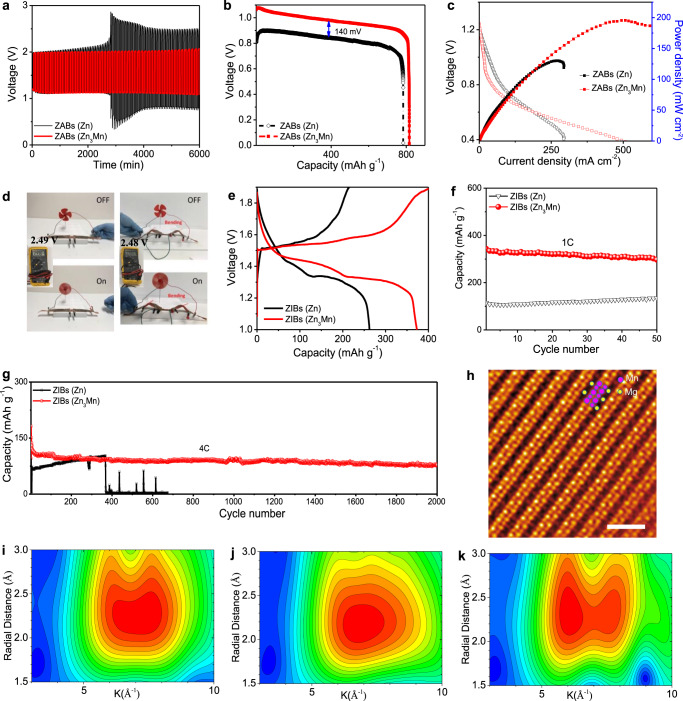
Electrochemical performance and characterizations of aqueous Zn batteries. **a** Cycling performance of ZABs (Zn_3_Mn) and ZABs (Zn). **b** Discharging plateaus of ZABs (Zn_3_Mn) and ZABs (Zn) at a current density of 30 mA cm^−2^. **c** Discharging and power density plots of ZABs (Zn_3_Mn) and ZABs (Zn). **d** Photograph of an electric fan powered by two flexible ZABs (Zn_3_Mn). **e** Typical charge/discharge profiles of ZIBs (Zn_3_Mn) at 0.5 C (electrolyte: 2 M ZnSO_4_ and 0.1 M MnSO_4_ in seawater). Cycling performance of ZIBs (Zn_3_Mn) at **f** 1C and **g** 4C, respectively. **h** High-resolution HAADF-STEM image of a fully discharged MnO_2_ cathode for ZIBs (Zn_3_Mn) using Mg^2+^-containing aqueous electrolyte. The yellow and pink dots represent Mg and Mn atoms, respectively. Scale bar: 1 nm. Wavelet transform of Mn K-edge EXAFS for **i** pristine Zn-Mn anode, **j** fully discharged Zn-Mn anode, and **k** fully charged Zn-Mn anode.

To demonstrate the practical performance of the Zn-Mn anode in aqueous batteries, we assembled ZABs using commercial Pt/C@RuO_2_ as the cathode and Zn-Mn alloy as the anode (Supplementary Fig. 34a). A control battery was assembled using the pristine Zn as the anode for a comparison. The ZABs using Zn-Mn anodes showed excellent charge/discharge cycling stability for over 6000 min test without degradation at a current density of 10 mA cm^−2^. In contrast, the ZABs using Zn anodes failed quickly after 2760 min test with a huge hysteresis (Fig. 4a). The galvanostatic discharge capacities of ZABs using different anodes were recorded (Fig. 4b and Supplementary Fig. 34b). Note that ZABs (Zn_3_Mn) and ZABs (Zn) are used to represent the batteries using Zn_3_Mn and Zn anodes, respectively, to reduce the wordy description. At a high current density of 30 mA cm^−2^, the ZABs (Zn_3_Mn) delivered an extremely high discharge capacity of 816.3 mAh g_Zn_^−1^ corresponding to an energy density of 798.3 Wh kg_Zn_^−1^, higher than those of ZABs (Zn; 784 mAh  g_Zn_^−1^ and 657 Wh kg_Zn_^−1^) and superior to the recent benchmarking ZABs^49–51^. The significantly improved performance of the ZABs (Zn_3_Mn) is ascribed to the sufficiently exposed active areas in the hierarchically porous 3D architectures via this surface/interface engineering^52^. To further demonstrate the outstanding ZABs (Zn_3_Mn) performance, we used our most recently developed materials composed of the co-incorporated platinum (Pt) and fluorine (F) in the PtCo nanosheets as a cathode to replace commercial Pt/C@RuO_2_^53^. As a proof-of-concept, a high peak power density of 196 mW cm^−2^ (Fig. 4c) was achieved by ZABs (Zn_3_Mn), which was much higher than that of ZABs (Zn) (130 mW cm^−2^). Besides, the Zn-Mn alloy is also mechanically robust and can be used for flexible ZABs. The flexible ZABs (Zn_3_Mn) in tandem cells exhibited nearly doubled voltages under different current densities. Under repeated twisting, the flexible tandem ZABs (Zn_3_Mn) retained a stable voltage and sustained an electric fan without any malfunction (Fig. 4d and Supplementary Movie 21). At the same time, the voltages of tandem ZABs (Zn_3_Mn) at high current densities were quite stable, confirming the outstanding performance for the Zn-Mn anode (Supplementary Fig. 35). Moreover, we assembled ZIBs full cells using MnO_2_ cathodes, Zn-Mn alloy anodes, and seawater-based electrolyte (2 M ZnSO_4_ and 0.1 M MnSO_4_ in seawater) to evaluate the electrochemical performance of Zn-Mn anode for aqueous ZIBs (Supplementary Fig. 36). The addition of Mn^2+^ in the electrolytes would improve the reversibility, greatly enhance the utilization of MnO_2_ active material, and suppress the dissolution of MnO_2_ in aqueous Zn//MnO_2_ batteries^2,54^. The ZIBs (Zn_3_Mn) using seawater-based electrolytes presented a higher capacity (373.2 mAh g^−1^, Fig. 4e) at 0.5 C and higher discharge voltage plateaus than that of ZIBs (Zn) (262.5 mAh g^−1^), confirming a more efficient charge transfer dynamics based on the Zn-Mn anode.

We also investigated the anti-interference property of Zn-Mn anode against hetero-ions such as Na^+^ and Mg^2+^ in the seawater-based electrolyte. As a control experiment, the ZIBs (Zn_3_Mn) using Na^+^-containing electrolyte (2 M Na_2_SO_4_ in seawater) showed a noticeable capacity of 30 mAh g^−1^, indicating a considerable storage capability in the ZIBs (Zn_3_Mn; Supplementary Fig. 37). Besides, we used a Mg^2+^-containing electrolyte (2 M MgSO_4_ in seawater) to test the Mg^2+^ anti-interference property in the ZIBs (Zn_3_Mn). A distinct intercalation behavior was observed in the ZIBs (Zn_3_Mn) with a high initial capacity of 110 mAh g^−1^ (Supplementary Figs. 38 and 39a, b) compared with the pristine Zn anode (Supplementary Fig. 40). We also investigate the impact of hetero-ions (Na^+^ and Mg^2+^) on the electrochemical performance of Zn-Mn alloy in the symmetric Zn-Mn//Zn-Mn cells (Supplementary Fig. 39c, d). And the anti-interference property of Zn-Mn anode against the other hetero-ions, including Ca^2+^ and Cl^−^, has also been investigated as shown in Supplementary Fig. 41, confirming the insignificant effect of hetero-ions (e.g., Ca^2+^ and Cl^−^) on the electrochemical performance of Zn-Mn alloy. The results also confirmed the highly anti-interference behaviors of the Zn-Mn anode. Furthermore, the ZIBs (Zn_3_Mn) using seawater-based electrolyte exhibited a stable capacity of 300 mAh g^−1^ at 1 C, whereas the ZIBs (Zn) delivered a much lower capacity of 130 mAh g^−1^ (Fig. 4f), demonstrating the superior electrochemical performance of ZIBs based on Zn-Mn anode in the seawater-based electrolyte. In particular, the self-discharge test of ZIBs (Zn_3_Mn) using the seawater-based electrolyte showed no drop in open-circuit voltage for 120 h (Supplementary Fig. 42). Furthermore, at a high rate of 4 C (Fig. 4g), the ZIBs (Zn) failed quickly after 368 cycles due to dendrite growth and short-circuit. In sharp contrast, the ZIBs (Zn_3_Mn) could keep a very stable performance over 2000 cycles without any dendrite growth and short-circuit (Supplementary Fig. 43), suggesting outstanding stability under harsh conditions far surpassing those of other benchmarking Zn anodes (Supplementary Table 3). The slow activation of the Zn//MnO_2_ batteries as shown in Fig. 4g could be caused by: (i) the diffusing paths of Zn^2+^ ion are gradually constructed due to the continuous infiltration of electrolytes after cycling; (ii) during the electrode activation process, more reactive sites could be exposed and the ionically conductive network of Zn^2+^ ion is greatly improved at the electrolyte/electrode interface^55,56^. To further demonstrate the broader impacts of the proposed concept in the battery field, we electrodeposited 3D Zn-Cu alloy (Supplementary Fig. 44), which could be another materials for high-performance aqueous batteries. Note that the 3D Zn-Cu anode is identified here as a potential extension of the proposed strategy for anode stabilization. We will fully discuss the battery performance of the Zn-Cu anode in our future work.

To understand the reaction mechanism and confirm the structural changes of the electrodes during the charge/discharge processes for ZIBs (Zn_3_Mn), we performed ex-situ X-ray absorption spectroscopy (XAS) measurements^53,57,58^ on the Zn-Mn anodes and MnO_2_ cathodes at pristine, fully charged, and fully discharged states. For MnO_2_ cathodes, we tested the intercalation behaviors of Zn-Mn/MnO_2_, which existed in the seawater-based electrolyte, by using the Mg^2+^-containing electrolyte. X-ray absorption near edge structure (XANES, Supplementary Fig. 45) on MnO_2_ cathode shows distinct edge shifts. At the fully discharged state for MnO_2_ cathode, the XANES spectrum at Mn K-edge moves to the lower energy compared to that of the pristine MnO_2_ cathode, suggesting the lower oxidation state of Mn and the successful intercalation of hetero-ions in the bulk structure. Furthermore, XANES of charged MnO_2_ cathode overlaps with the pristine one, implying good reversibility during charge/discharge processes. We also performed the HAADF-STEM analysis for the fully discharged MnO_2_ cathode, as shown in Supplementary Fig. 46. The low magnification image (Supplementary Fig. 46a) shows that the MnO_2_ nanowire maintained a good structure with a smooth surface, indicating the stability of the MnO_2_ cathode during the charge/discharge process. From the atomic resolution HAADF-STEM images (Fig. 4h and Supplementary Fig. 46b), Mg^2+^-insertion was observed in the structure of MnO_2_. These spectroscopic and microscopic characterizations suggested a completely reversible storage capability of hetero-ions (e.g. Mg^2+^) in MnO_2_ cathodes of ZIBs (Zn_3_Mn). The ex-situ XPS spectra of the cycled Zn-Mn alloy-based electrodes in Mg- and Na-containing seawater-based electrolytes were characterized as shown in Supplementary Fig. 47. XPS spectra demonstrate that the existence of the adsorption and/or binding of Mn with cations/metal^59,60^. To further confirm the local structure change on the Zn-Mn anode during charge/discharge processes, the one-dimensional (1D) Fourier transform of the extended X-ray absorption fine structure (EXAFS) spectra of Mn K-edge for the Zn-Mn anode at three states of charge/discharge process (Supplementary Fig. 48) was first applied. Although some changes are found, this 1D EXAFS does not have a good resolution to distinguish the broad peak ~2.2 Å^61,62^. Then the two-dimensional (2D) wavelet transform of the EXAFS spectra was used, as shown in Fig. 4i–k^58^. Clearly, the 2D spectra that combine the R-space and the k-space can distinguish the differences in three states. The single peak found in pristine (Fig. 4i) and fully discharged (Fig. 4j) Zn-Mn anode suggests the existence of Mn-Mn scattering only. In contrast, the fully charged (Fig. 4k) Zn-Mn anode has two well-splitting peaks, suggesting the co-existence of Mn-Mn and newly formed Mn-X (X = Mg, etc.) scattering that could be due to the adsorption and/or alloying of Mn with cations/metal. All these characterizations confirmed the success of the rationally designed Zn-Mn alloy anode and the benefits of using seawater-based electrolytes for aqueous Zn batteries.

## Discussion

In conclusion, we report a universal strategy for designing 3D Zn-Mn alloy anodes with a potential extension to other alloy-based anode materials for stable, high-performance, dendrite-free, seawater-based aqueous batteries. Equally important, we built an in-situ protocol to mimic the actual electrochemical environments of aqueous batteries and directly observe the metal plating/stripping processes on the electrode surface. The 3D Zn-Mn alloy anode, even under harsh electrochemical environments (hetero-ions interference from the seawater-based electrolyte and high current density of 80 mA cm^−2^), maintained controllable Zn plating/stripping with robust structural stability and absolute reversibility for aqueous batteries. As a proof-of-concept, the seawater-based aqueous ZIBs and ZABs using Zn-Mn alloy anodes delivered outstanding performance towards energy storage, which proved the novelty and significance of this work. The concept demonstrated in this work will bring a paradigm shift in the design of high-performance alloy anodes for aqueous/non-aqueous batteries and beyond, therefore, revolutionizing the battery industries.

## Methods

### Galvanostatic alloy electrodeposition of Zn-Mn alloys

All three-dimensional (3D) structured Zn-Mn alloys were electrodeposited on Zn substrates (99.95% metals basis, 0.25 mm thick, Alfa Aesar^TM^). In all, 100 mL deionized (DI) water was pre-heated at 80 °C as the solvent to dissolve 0.2 M zinc sulfate heptahydrate (ZnSO_4_·7H_2_O, Fisher Chemical), 0.2 M sodium citrate dihydrate (Granular/Certified), and 0.6 M ethylenediaminetetraacetic acid disodium salt dihydrate (Crystalline/Certified ACS, Fisher Chemical) under continuous stirring for 30 min (noted as Solution A). Then, 0.6 M manganese (II) sulfate monohydrate (MnSO_4_·H_2_O, 99+%, extra pure, ACROS Organics™) was added to Solution A and stirred for another 30 min until a transparent solution was obtained (noted as Solution B). The Zn-Mn alloys were then deposited on Zn substrates using a two-electrode setup with platinum mesh as the counter electrode at a current density of 0.3 A cm^−2^ in Solution B.

### Potentiostatic alloy electrodeposition of Zn-Cu alloys

In total, 100 mL DI water was pre-heated as the solvent to dissolve zinc sulfate heptahydrate (ZnSO_4_·7H_2_O, Fisher Chemical), copper (II) sulfate pentahydrate (Fisher Chemical), and boric acid (Powder/Certified ACS, Fisher Chemical) under continuous stirring for 20 min until a transparent solution was obtained (noted as Solution C). The Zn-Cu alloys were deposited on Zn substrates using the two-electrode setup in Solution C.

### Zn@Zn anode fabrication

The Zn@Zn anode was electrodeposited in Solution A using the same conditions as those for the deposition of Zn-Mn alloy.

### Seawater-based aqueous electrolytes

Nine kinds of aqueous electrolytes were prepared: Electrolyte 1 (2 M ZnSO_4_ and 0.1 M MnSO_4_ in DI water); Electrolyte 2 (2 M ZnSO_4_ in DI water); Electrolyte 3 (2 M ZnSO_4_ and 0.1 M MnSO_4_ in seawater); Electrolyte 4 (2 M ZnSO_4_ in seawater); Electrolyte 5 (1 M ZnSO_4_ and 1 M MgSO_4_ in seawater); Electrolyte 6 (1 M ZnSO_4_ and 1 M MgSO_4_ in DI water); Electrolyte 7 (2 M MgSO_4_ in seawater); Electrolyte 8 (2 M Na_2_SO_4_ in seawater); and Electrolyte 9 (2 M MgSO_4_ in DI water). The seawater was taken from Florida’s nearshore zone, physically filtered to remove the suspended particles, and directly used in this work without any other treatment.

### Cathode preparation for rechargeable Zn aqueous batteries

MnO_2_ cathode materials were prepared for Zn-ion batteries (ZIBs) full-cell testing by a hydrothermal method. Typically, 0.5 g MnSO_4_·H_2_O and 2 mL 0.5 M H_2_SO_4_ were added to 100 mL DI water under continuous stirring until a clear solution (noted as Solution D) was obtained. After that, 25 mL 0.1 M KMnO_4_ aqueous solution was slowly added to Solution D and stirred for 5 h. The as-prepared solution was transferred to a Teflon-lined PTFE autoclave vessel and heated at 120 °C for 8 h. Then, MnO_2_ powder was collected, washed by DI water, and dried at 60 °C overnight in a vacuum oven. The ZIBs cathodes were prepared by a doctor-blade method. First, MnO_2_ powder, polyvinylidene fluoride (PVDF) binder, and super P carbon were mixed in N-methyl pyrrolidinone (NMP) solvent in a weight ratio of 7:1:2 to get a homogenous slurry. Then, the obtained mixed slurry was coated onto carbon paper (CP) and dried at 80 °C overnight in the vacuum oven.

Pt/C@RuO_2_ and F-doped PtCo nanosheets on the nickel foam (PtCoF@nickel foam) were prepared as cathodes for Zn-air batteries (ZABs) testing according to our prior work^53^. The Pt/C@RuO_2_ cathode was prepared in the following procedure: (1) 3.2 mg Pt/C powder was mixed with 3.2 mg RuO_2_ in the 3.2-ml Nafion/isopropanol solution (98:2, v/v), and then ultrasonicated for 20 min. The obtained suspension was disposed on 4 × 4 cm^2^ carbon paper and dried at 60 °C. The single-atom PtCoF@nickel foam was prepared by fluorine (F)-plasma treatment using carbon tetrafluoride as a source in a plasma etcher (Trion MiniLock II RIE-ICP) using the PtCo@nickel foam as a precursor.

### Electrochemical tests

Symmetric cells were assembled using Zn (or Zn-Mn alloy) foils as both cathode and anode, which were separated by a glass fiber membrane saturated with different aqueous electrolytes. For Cu//Zn (or Cu//Zn-Mn) cells, Cu and Zn (or Zn-Mn alloy) foils were used as cathode and anode, respectively, for the plating/stripping tests in the aqueous Zn batteries. The active areas of electrodes were 1 cm^2^ (1 cm × 1 cm) in coin cells. Cyclic voltammetry (CV) and electrochemical impedance spectroscopy (EIS) data were measured by CHI 600E electrochemical workstation. The electrochemical performance of aqueous electrolytes was tested in a three-electrode setup (Pt mesh as the working electrode, Zn (or Zn-Mn alloy) foil as both counter and reference electrodes) at a scan rate of 1 mV s^−1^.

Zn (or Zn-Mn alloy) anodes and MnO_2_@Carbon Paper (MnO_2_@CP) cathodes were assembled in CR2032 coin cells for the ZIBs full-cell testing. The mass loading of MnO_2_ was 2–3 mg cm^−2^. Pt/C@RuO_2_ (or PtCoF@nickel foam) cathodes and Zn (Zn-Mn alloy) anodes were assembled with an electrolyte consisting of 6 M KOH and 0.2 M zinc acetate for ZABs full-cell testing. Gel electrolytes were also prepared by mixing polyvinyl alcohol (PVA) powder with 6 M KOH and 0.2 M zinc acetate at 80 °C to assemble the flexible ZABs.

### Materials characterizations

X-ray diffraction patterns (XRD) were obtained on a film XRD system (Panalytical X’celerator multi-element detector with Cu Kα radiation source, λ = 1.54056 Å). The surface topographies were characterized by atomic force microscopy (AFM, Veeco Dimension 3100) using tapping mode. The contact angles were measured with an OCA 15EC goniometer and analyzed with the SCA 20 module from DataPhysics Instruments. A droplet volume of 3 µL was used for each measurement. The morphologies of the materials were characterized by scanning electron microscopy (SEM, ZEISS ultra 55) with EDS mapping. Transmission electron microscopy (TEM), high angle annular dark-field scanning transmission electron microscopy (HAADF-STEM), and X-ray spectroscopy (EDS) were performed using a probe corrected FEI Titan 80–300 microscope operating at 300 kV. Mn K-edge X-ray absorption spectroscopy experiments were carried out at beamline 12BM, Advanced Photon Source (APS), Argonne National Laboratory (ANL). Data reduction, data analysis, and EXAFS fitting were performed with the Athena, Artemis, and IFEFFIT software packages.

### In-situ optical imaging

To realize in-situ imaging of Zn plating/stripping dynamics, a two-electrode system was used in which the pristine Zn foils were employed as counter and reference electrodes, and 3D Zn-Mn alloy was used as a working electrode. To realize the in-situ imaging in the aqueous electrolyte, a special electrochemical cell was designed using polydimethylsiloxane (PDMS, prepared by the mixing of base elastomer and curing agent with a ratio of 10:1 and then cross-linking for 3 h at 75 °C) to hold the electrolyte. In all, 800 μL electrolyte was applied to the cell with a size of 1 cm × 1 cm × 3 mm. Images were recorded with a CCD camera (FLIR Blackfly S USB 3, 720 × 540 pixels) on an Olympus BX60 upright microscope. To minimize the refractive index mismatch between the air and high concentration saline electrolyte, a ×20 water immersion objective (working distance: 2 mm, N.A. 1.0, Thorlab) was submerged into the electrolytes and the reflected images of the electrode surface were obtained. The setup diagram is shown in Fig. 3a. The imaging area was 200–400 μm away from the top electrode’s projection. Different current densities (5–80 mA cm^−2^) were applied, and four electrolytes (Electrolyte 1: 2 M ZnSO_4_ in seawater; Electrolyte 2: 2 M ZnSO_4_ with 0.1 M MnSO_4_ in seawater; Electrolyte 3: 2 M ZnSO_4_ with 0.1 M MnSO_4_ in DI water; Electrolyte 4: 2 M ZnSO_4_ in DI water) were tested to investigate the Zn plating process. To compare with the 3D Zn-Mn alloy, a pristine Zn foil anode was also tested in Electrolyte 2. After the Zn plating, the current density of 80 mA cm^−2^ was used to analyze the stripping process of the 3D Zn-Mn anode.

### DFT calculations

Density functional theory (DFT) simulation was conducted to analyze the adsorption and kinetics of Zn ad-atoms on the experimentally confirmed Zn_3_Mn (110) surface. For the simplicity and the efficiency of the calculation, the simulation was conducted on the cubic cell to extract the effect of Mn substitution alone. The simulation model was constructed with 40 Zn and 16 Mn atoms in a unit cell of 1.08 × 0.76 × 2.08 nm. The *x*, *y*, and *z* directions of the cell correspond to [001], [1–10], and [110] crystal orientation, respectively. A vacuum layer of 1.2 nm was included in the *z*-direction to avoid the interaction from the neighboring cells in a periodic boundary condition. Vienna Ab-initio Simulation Package (VASP) was used in the calculation^63,64^, with projector augmented wave (PAW) pseudopotential^65,66^ and generalized gradient approximation by Perdew-Burke-Ernzerhof^67^. The plane wave energy cut off was 400 eV and 3 × 5 × 1 *k*-points were selected based on the Monkhorst-Pack method^68^. First, the conjugate gradient structure optimization was performed while fixing the atoms in the bottom two layers. Then, a Zn atom was placed on the surface at 10 × 10 grid points and structural optimization was performed. Here only the ad-atom was relaxed in *z*-direction while fixing the *x* and y coordinates. Also, a Zn ad-atom was placed on a surface lattice point and structure optimization was conducted to calculate the binding energy. The binding energy calculation was also performed on the Zn (110) surface for comparison. The binding energies were calculated by the (total energy of the system w/o Zn ad-atom) + (isolated Zn atom) – (total energy of the model w/ Zn ad-atom).

## Supplementary information

Supplementary Information

Description of Additional Supplementary Files

Supplementary Movie 1

Supplementary Movie 2

Supplementary Movie 3

Supplementary Movie 4

Supplementary Movie 5

Supplementary Movie 6

Supplementary Movie 7

Supplementary Movie 8

Supplementary Movie 9

Supplementary Movie 10

Supplementary Movie 11

Supplementary Movie 12

Supplementary Movie 13

Supplementary Movie 14

Supplementary Movie 15

Supplementary Movie 16

Supplementary Movie 17

Supplementary Movie 18

Supplementary Movie 19

Supplementary Movie 20

Supplementary Movie 21

## Data Availability

The data that support the findings of this study are available from the corresponding author upon reasonable request.
