# Supplementary material for: Stable, high-performance, dendrite-free, seawater-based aqueous batteries
Source: Nat Commun. 2021 Jan 11;12:237. doi: 10.1038/s41467-020-20334-6 (PMC7801520; doi:10.1038/s41467-020-20334-6)
Supplement: Supplementary file 1 — Supplementary Information [file 41467_2020_20334_MOESM1_ESM.pdf]

Supplementary Information for

Stable, High-performance, Dendrite-free, Seawater-based Aqueous Batteries

Huajun Tian[1], Zhao Li[1], Guangxia Feng[2], Zhenzhong Yang[3], David Fox[1,4], Maoyu Wang[5], Hua Zhou[6], Lei Zhai[1,4], Akihiro Kushima[1,7,8], Yingge Du[3], Zhenxing Feng[5,*], Xiaonan Shan[2,*], Yang Yang[1,7,9,*]

[1] NanoScience Technology Center, University of Central Florida, Orlando, Florida 32826, USA

[2] Electrical and Computer Engineering Department, W306, Engineering Building 2, University of Houston, Houston, TX 77204, USA

[3] Physical and Computational Sciences Directorate, Pacific Northwest National Laboratory, Richland, Washington 99352, USA

[4] Department of Chemistry, University of Central Florida, Orlando, Florida 32826, USA

[5] School of Chemical, Biological, and Environmental Engineering, Oregon State University, Corvallis, OR 97331, USA

[6] X-ray Science Division, Argonne National Laboratory, Lemont, IL 60439, USA

[7] Department of Materials Science and Engineering, University of Central Florida, Orlando, Florida 32826, USA

[8] Advanced Materials Processing and Analysis Center, University of Central Florida, Orlando, Florida 32826, USA

[9] Energy Conversion and Propulsion Cluster, University of Central Florida, Orlando, Florida32826, USA

Corresponding Author

[*]E-mail: zhenxing.feng@oregonstate.edu; xshan@central.uh.edu; Yang.Yang@ucf.edu

Supplementary Discussions

1. *Growth mechanism of 3D Zn-Mn alloy by electrodeposition*

The Stranski-Krastanov growth mechanism (**Supplementary Fig. 1**) was suggested to explain the formation of 3D Zn-Mn alloy through heterogeneous nucleation and growth processes. In detail, the electrodeposition process includes heterogeneous chemical reactions at the liquid-solid interface. In the initial stage, the nucleation and growth processes were accompanied by the diffusion of highly mobile clusters or islands of the deposits on the electrode surface. In the following stage, the nuclei coalesced to form a continuous film with 3D structures on the top (**Supplementary Fig. 2**).

2. *Surface wettability of 3D structured and alloyed electrodes*

3D structured Zn electrode was prepared to compare the surface wettability with the 3D Zn_3Mn alloy electrodes. The surface wettability of the 3D Zn_3Mn electrode was demonstrated to be much more superhydrophilic than the 3D structured Zn electrode. We also found that the nanostructured 3D surface played a dominant role in achieving a superhydrophilic surface, while the chemical composition determined the intrinsic wettability of the electrodes. Firstly, we examined the effect of surface morphology (roughness) on wettability by measuring the contact angle of the water droplet on the electrode surface according to Young's equation:[1]

$$\gamma_{sv} = \gamma_{sl} + \gamma_{lv} cos\theta$$

where θ is the contact angle, γ is the surface tension at the interfaces of different phases of solid (s), liquid (l), and vapor (v). A superhydrophobic and superhydrophilic surface usually shows a contact angle > 150° and < 5°, respectively, which is determined by the surface roughness. The Wenzel contact angle (θ^W) describes the wetting behavior of the electrode surface affected by the roughness (r) according to the r-θ^W relationship expressed by the Wenzel equation, $cos\theta^W = r\, cos\theta$. The higher surface roughness, the more hydrophilic surface. This well-established roughness-dependent wetting behavior is also applicable to the 3D structured Zn electrodes. The liquid may sink partially into the 3D structured Zn surface; however, it likely does not experience capillary forces that draw it deep further into the 3D surface, leading to a less hydrophilic surface.

For the 3D structured Zn_3Mn electrode, a superhydrophilic wetting was observed, where water droplets could deeply penetrate the 3D surface of the Zn_3Mn electrode. The liquid pulled

into the water-permeable surface of the 3D electrode increases the affinity of the liquid droplet with the electrode surface, resulting in a more superhydrophilic surface. As these materials exhibit such a high water affinity and likely can pull water into the electrode through nanocapillary forces,[2] this may provide unique merits for energy applications of 3D Zn_3Mn electrodes in aqueous electrolyte systems.

Both the intrinsic wettability of the electrodes determined by the chemical composition and the nanostructural effect should be considered. The commercial Zn foils exhibit various contact angles,[3] due to the uncontrollable manufacturing defects. On the other hand, the commercial metal alloys such as AZ31 Mg alloy (Mg:96/Al:3/Zn:1) show a stable contact angle,[4] which has less dependence on the manufacturing process. Thereupon, the 3D Zn_3Mn alloy electrode shows a stable superhydrophilicity benefited from both 3D structure and Zn_3Mn alloyed composition.

3. <u>COMSOL simulations</u>

Multiple two-dimensional (2D) and three-dimensional (3D) COMSOL models (COMSOL Multiphysics 5.5a) were established to understand the dynamics of the electrochemical processes. The Zn plating process will change the thickness and profile of the electrode. To simulate this process, the deformed mesh interface was employed in the COMSOL models. The boundary conditions were given by the Bulter-Volmer equation for Zn plating and the local current density was a function of potential and Zn^{2+} concentration:

$$i_{ct} = i_0(\exp\left(\frac{1.5F\eta}{RT}\right) - \frac{C_{Zn^{2+}(t)}}{C^*_{Zn^{2+}}}\exp\left(-\frac{0.5F\eta}{RT}\right)) \quad (1)$$

where η denotes the overpotential (V), F Faraday's constant (As/mole), $C^*_{Zn^{2+}}$ initial Zn^{2+} concentration (mol m^{-3}) and i_0 the initial current density (A m^{-2}). The initial current density has been calculated with the actual experimental conditions (80 mA cm^{-2}). The Bulter-Volmer equations for the cathode and anode are listed below:

$$N_{Zn^{2+}} * n = -\frac{i_0}{2F}(\exp\left(\frac{1.5F\eta_{cathode}}{RT}\right) - \frac{C_{Zn^{2+}(t)}}{C^*_{Zn^{2+}}}\exp\left(-\frac{0.5F\eta_{cathode}}{RT}\right)) \quad (2)$$

$$N_{Zn^{2+}} * n = -\frac{i_0}{2F}(\exp\left(\frac{1.5F\eta_{anode}}{RT}\right) - \frac{C_{Zn^{2+}(t)}}{C^*_{Zn^{2+}}}\exp\left(-\frac{0.5F\eta_{anode}}{RT}\right)) \quad (3)$$

The related Zn^{2+} concentration, current density, and all other parameters were defined and listed in **Supplementary Table 4**.

4. <u>2D COMSOL model to simulate the Zn plating</u>

The dimension of the electrochemical cell was 3 mm × 10 mm. The top and bottom boundaries were set as the cathode and anode (**Model 1**), respectively. To simulate the Zn-Mn structures, hundreds of semi-circles with a radius of 10 µm were added to the bottom electrode (**Supplementary Fig. 26a**). The current density on the bottom electrode was set to be 800 A m^{-2} (80 mA cm^{-2}, **Supplementary Fig. 26b**) to match the actual experimental conditions. In **Supplementary Fig. 26c**, the dashed lines show the observation area of the optical *in-situ* microscope. Note that the edge of the top electrode was at 3 mm location on the x-axis. The current at the observation area maintains 95% of the maximum current, demonstrating the feasibility of *in-situ* visualization for electrochemical reactions.

5. *2D COMSOL model to simulate the Zn plating in trenches*

To have an insightful understanding of the Zn plating process on the Zn-Mn alloy, a minimized COMSOL model (**Supplementary Fig. 30a**, **Model 3**) was built. On the bottom electrode, three semi-circles with a radius of 10 µm were used to mimic the Zn-Mn structures. **Supplementary Figs. 30b-d** show the electrode morphology change caused by 30s of Zn plating and the colors represent the current density distribution. The average deposition rate over the 30s was calculated. As shown in **Supplementary Fig. 25a**, the trench had a higher plating rate than the other regions, confirming our experimental observation in **Fig. 3**.

To further mimic the actual Zn-Mn alloy, nano-voids were added on the semi-circles to simulate the porous structure (**Supplementary Fig. 28a**, **Model 2**). **Supplementary Figs. 28b-d** show the electrode morphology change caused by 10s of Zn plating and the colors represent the current density. The average plating rate was calculated (**Supplementary Fig. 25b**) to show that the nano-voids and trench had a much higher deposition rate. It indicates that the nano-voids will be filled first as the reaction proceeds.

We also simulated the Zn deposition up to 200s using the established COMSOL model and calculated the deposition rate in the trench and on the protruding regions (on top of the semi-circles) throughout the entire plating process. As shown in **Supplementary Fig. 25c**, the Zn deposition rate inside the trench (black line) is much higher than that (red line) of the protruding region (on top of the semi-circle) at the beginning of the deposition. However, as the plating continued, the deposition rate in the trench decreased, and eventually approached to that of the protruding region.

This will lead to a uniform electrode after Zn deposition, which further demonstrates the capability of our 3D Zn-Mn structures to suppress the dendrite formation.

6. *3D COMSOL model to simulate the Zn plating*

To understand the Zn plating dynamics in the aqueous battery cell, we established a 3D COMSOL model. As shown in **Supplementary Fig. 24**, the areas of the top and bottom electrodes were 61 μm × 61 μm, and a series of semi-spheres with the radius of 10 μm was designed to mimic the 3D Zn-Mn alloy structures and the distance between the top and bottom electrodes is 100 μm. **Fig. 2e and Fig. 2f** show the morphology of the bottom electrode before and after 50s deposition, respectively. **Fig. 2g** shows the morphology change during the 50s Zn plating. We can easily conclude that the trench has a much higher Zn deposition rate, which is consistent with our *in-situ* experimental and the 2D COMSOL results. The simulation result confirms that the 3D Zn-Mn alloy will intrinsically suppress the dendrite growth for high-performance aqueous Zn batteries.

7. *In-situ optical imaging of Zn stripping*

For the *in-situ* observation of the Zn stripping process, we firstly performed Zn plating on the 3D Zn-Mn alloy for 160s with the current density of 80 mA cm^{-2}. Then, under the same experimental condition, we conducted the stripping process for 240s with the current density of 80 mA cm^{-2}. As shown in **Supplementary Fig. 31**, when the plating happened, the trenches were filled up first, and the whole surface became smooth eventually. After the stripping process, the original structures on the electrode almost completely recovered. This *in-situ* Zn plating/stripping imaging results strongly support our conclusion that the 3D Zn-Mn alloy can serve as ideal metal anodes for stable, high-performance, dendrite-free, seawater-based aqueous batteries.

Supplementary Tables

Supplementary Table 1. Reduction potentials of different metal ions in reduction half-reaction.

Reduction half-reaction	Reduction potential
Zn^{2+} (aq) + $2e^{-}$ → Zn (s)	-0.76 V
Mn^{2+} (aq) + $2e^{-}$ → Mn (s)	-1.18 V
Mg^{2+} (aq) + $2e^{-}$ → Mg (s)	-2.37 V
Al^{3+} (aq) + $3e^{-}$ → Al (s)	-1.66 V
Co^{2+} (aq) + $2e^{-}$→Co (s)	-0.28 V
Ni^{2+} (aq) + $2e^{-}$ → Ni (s)	-0.25 V
Cu^{2+} (aq) + $2e^{-}$ → Cu (s)	+0.34 V

Supplementary Table 2. The cost comparison of the seawater and commercial solvents for aqueous batteries (Note: the prices may have variations depending on the market).

Solvent	Supplier	Pack Size	Price
Water (HPLC grade)	Fisher Scientific™	1L	$70.00
Water (HPLC grade)	Sigma-Aldrich	1L	$52.00
Water (HPLC grade)	Alfa Aesar	1L	$36.70
DI water	Sigma-Aldrich	1L	$22.00
DI water	Alfa Aesar	1L	$26.4
DI water	Fisher Scientific™	1L	$21.90
**Seawater (in this work)**	**Florida's nearshore zone (near UCF)**	**Almost zero cost; directly used in this work after removing the suspended particles by an inexpensive filter paper**	

Supplementary Table 3. Electrochemical performance of state-of-the-art Zn metal-based anodes for aqueous Zn batteries.

Anode	Electrolyte	J_{max} (mA cm^{-2})	Stability	Ref.
**3D Zn-Mn alloy**	**2 M $ZnSO_4$ in Seawater**	**80**	**1900 cycles, 760 h**	**This work**
Tin (Sn)-modified 3D carbon felt@Zn	2 M $ZnBr_2$, 3 M KCl, and 0.8 M N-methylethylpyrrolidinium bromide in DI water	40	290 h	[5]
3D Zn sponge	6 M KOH in DI water	24	80 h	[6]
Zn foam	1 M $ZnSO_4$ + 1 M $MnSO_4$ with 0.1M H_2SO_4 in DI water	20	100 h	[7]
Polyamide coated Zn	2 M $ZnSO_4$ in DI water	10	150 h	[8]
Zn/rGO	1 M $ZnSO_4$ in DI water	5	80 h	[9]
Zn powder-coated on Al foil	1m $Zn(TFSI)_2$ + 20m LiTFSI in DI water	0.2	170 h	[10]
Zn foil	Water@ZnMOF-808 (WZM) solid electrolyte	0.1	360 h	[11]
Zn/CNT	2 M $ZnSO_4$ in DI water	5	110 h	[12]
Zn/SS mesh	3 M $Zn(CF_3SO_3)_2$ in DI water	2	300 h	[13]
Zn@ZIF-8	2 M $ZnSO_4$ in DI water	1	50 h	[14]
Zn@porous kaolin	2 M $ZnSO_4$ + 0.1 M $MnSO_4$ in DI water	4.4	800 h	[15]
Zn plate@TiO_2	3 M $Zn(CF_3SO_3)_2$ in DI water	1	150 h	[16]
Zn@HsGDY	2 M $ZnSO_4$ in DI water	2	2400 h	[17]
Zn@PVB polymer	1 M $ZnSO_4$ in DI water	0.5	2200 h	[18]
Zn@porous nano-$CaCO_3$	3 M $ZnSO_4$ + 0.1 M $MnSO_4$ in DI water	0.25	836 h	[19]
Zn@carbon fibers(CFs)	2 M $ZnSO_4$ + 0.1 M $MnSO_4$ in DI water	1	160 h	[20]
Porous copper skeleton supported Zn	2 M $ZnSO_4$ in DI water	0.5	350 h	[21]
$Ti_3C_2T_x$ MXene@Zn paper	2 M $ZnSO_4$ in DI water	1	300 h	[22]
Cu foam@Zn	2 M $ZnSO_4$ + 0.1 M $MnSO_4$ in DI water	2	150 h	[23]
Zr coated Zn	2 M $ZnSO_4$ in DI water	5	2100 h	[24]
Zn/PAN	2 M $ZnSO_4$ in DI water	0.5	350 h	[25]
CNTs coated Zn	2 M $ZnSO_4$ + 1 M $MnSO_4$ in DI water	0.5	400 h	[26]

Zinc-plated copper mesh	1 M $ZnSO_4$ + 0.5 M Na_2SO_4 + 1 g L^{-1} Polyacrylamide (PAM) in DI water	0.2	350 h	[27]
Active carbon coated zinc foil	8 M $NaClO_4$ + 0.4 M $Zn(CF_3SO_3)_2$ in DI water	1	100 h	[28]
Carbon fiber-graphite felt	0.5 M $ZnSO_4$ + 0.5 M Na_2SO_4 in DI water	1	700 h	[29]
Eutectic $Zn_{88}Al_{12}$	2 M $ZnSO_4$ + 0.2 M $MnSO_4$ in DI water	0.5	400 h	[30]
Zn/Reduced graphene oxide	0.5 M $ZnSO_4$ in DI water	1	300 h	[31]
Copper foam@Zn	2 M $ZnSO_4$ + 0.1 M $MnSO_4$ in DI water	2	300 h	[32]
Mesoporous hollow carbon spheres coated Zn foil	2 M $ZnSO_4$ in DI water	1	500 cycles	[33]
MOF-PVDF coated Zn foil	3 M $ZnSO_4$ + 0.1 M $MnSO_4$ in DI water	3	100 h	[34]
3D Zn array	2 M $ZnSO_4$ with 4% fumed silica in DI water	1	280 cycles	[35]
Zinc foil	$Zn(TFSI)_2$/acetamide eutectic solution, m/m=1:4 and 1:9	1	100 h	[36]
Zinc foil	LiTFSI+ $Zn(TFSI)_2$+ urea in DI water	0.02	400h	[37]
Zinc foil	3M $Zn(CF_3SO_3)_2$ + diethyl ether (2 vol.%) in DI water	0.2	250 h	[38]
Zinc foil	30 m $ZnCl_2$ in DI water	0.2	600 h	[39]
Zinc foil	$Zn(ClO_4)_2$ in DI water	2	1100 h	[40]
Zinc foil	Gelatin powder+1 M $ZnSO_4$ + 0.1 M $MnSO_4$ in DI water	5	400 h	[41]
Zinc foil	$ZnSO_4$+$MnSO_4$ +FS+FMEE (1 M+0.01 M) in DI water	0.2	1500 h	[42]
Zinc foil	0.5 M $Zn(CF_3SO_3)_2$ in (TEP-H_2O)	0.25	1000 h	[43]
Zinc foil	3 M $ZnSO_4$ in DI water	0.1	50 h	[44]
Zinc foil	3 M $ZnSO_4$ + 0.1 M $MnSO_4$ in DI water	0.1	180 h	[45]

Supplementary Table 4. Concentration and related parameters.

Name	Expression	Value	Description
Cinit	2000[mol/(m^3)]	2000 mol/m^3	Initial concentration
T0	298[K]	298 K	System temperature
i0_ref	15[A/m^2]	15 A/m^2	Exchange current density
phis_anode	0.135[V]	0.135 V	Anode potential
phis_cathode	-0.135[V]	−0.135 V	Cathode potential
alpha_c	0.5[1]	0.5	Symmetry factor
alpha_a	1.5[1]	1.5	Symmetry factor
z_c1	z_net[1]	2	Charge, species c1
z_c2	-z_net[1]	−2	Charge, species c2
D_c1	2e-9[m^2/s]	2E−9 m^2/s	Diffusivity, species c1

Supplementary Figures

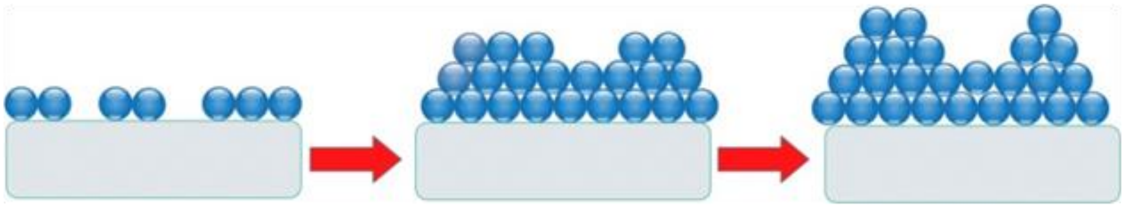

Supplementary Figure 1. Illustration of the suggested Stranski-Krastanov growth process of 3D Zn-Mn alloy.

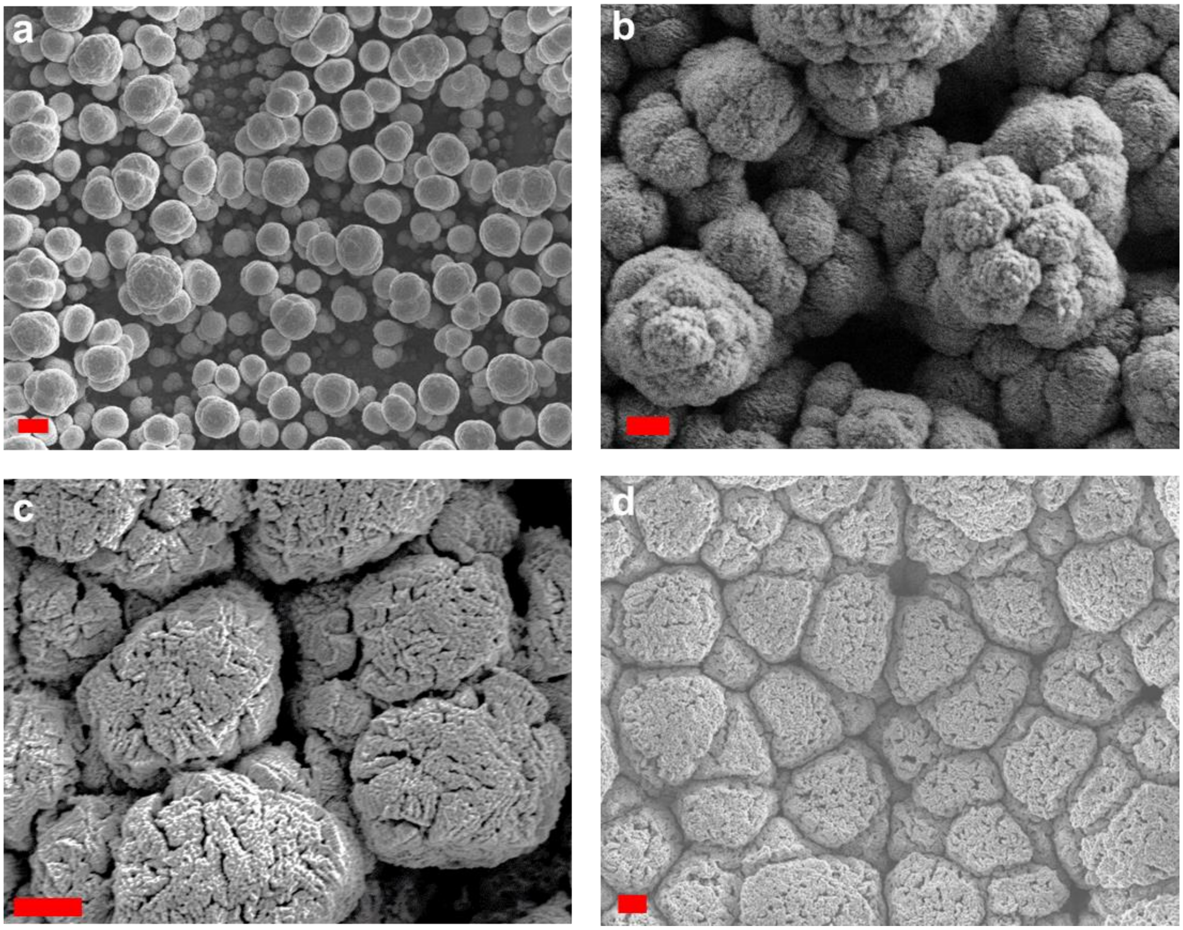

Supplementary Figure 2. Growth of Zn-Mn alloy. SEM images of Zn-Mn alloy electrodeposited for (a) 10 min, (b) 20 min, (c) 30 min, and (d) 40 min. Scale bars: 10 μm. The Zn-Mn alloy clusters or islands initially nucleated and then coalesced to form a continuous film. Meanwhile, a large number of pores formed in the 3D alloy due to the evolution of hydrogen bubbles.

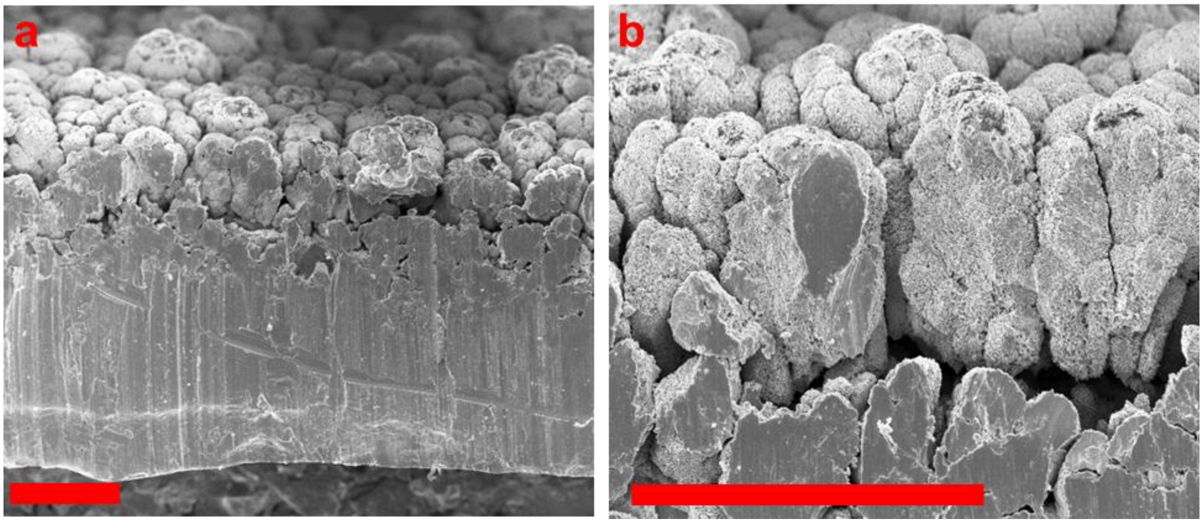

Supplementary Figure 3. Morphologies of Zn-Mn alloy. The cross-sectional SEM images of (a) Zn-Mn alloy@Zn and (b) Zn-Mn alloy. Scale bars: 100 μm. Cauliflower-shaped Zn-Mn alloy firmly anchors on the Zn substrate surface. Many hierarchical pores were observed.

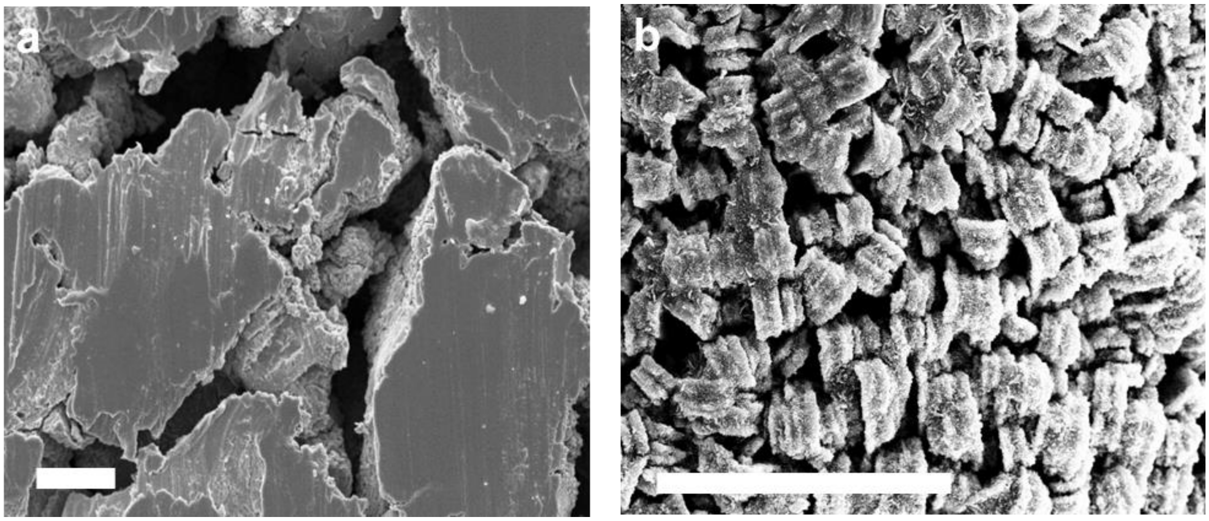

Supplementary Figure 4. Morphologies of as-prepared Zn-Mn alloy. (a) Cross-sectional and (b) top-view SEM images of Zn-Mn alloy prepared by 40 min deposition. Scale bars: 10 μm. A porous structure was formed by the evolved hydrogen bubbles.

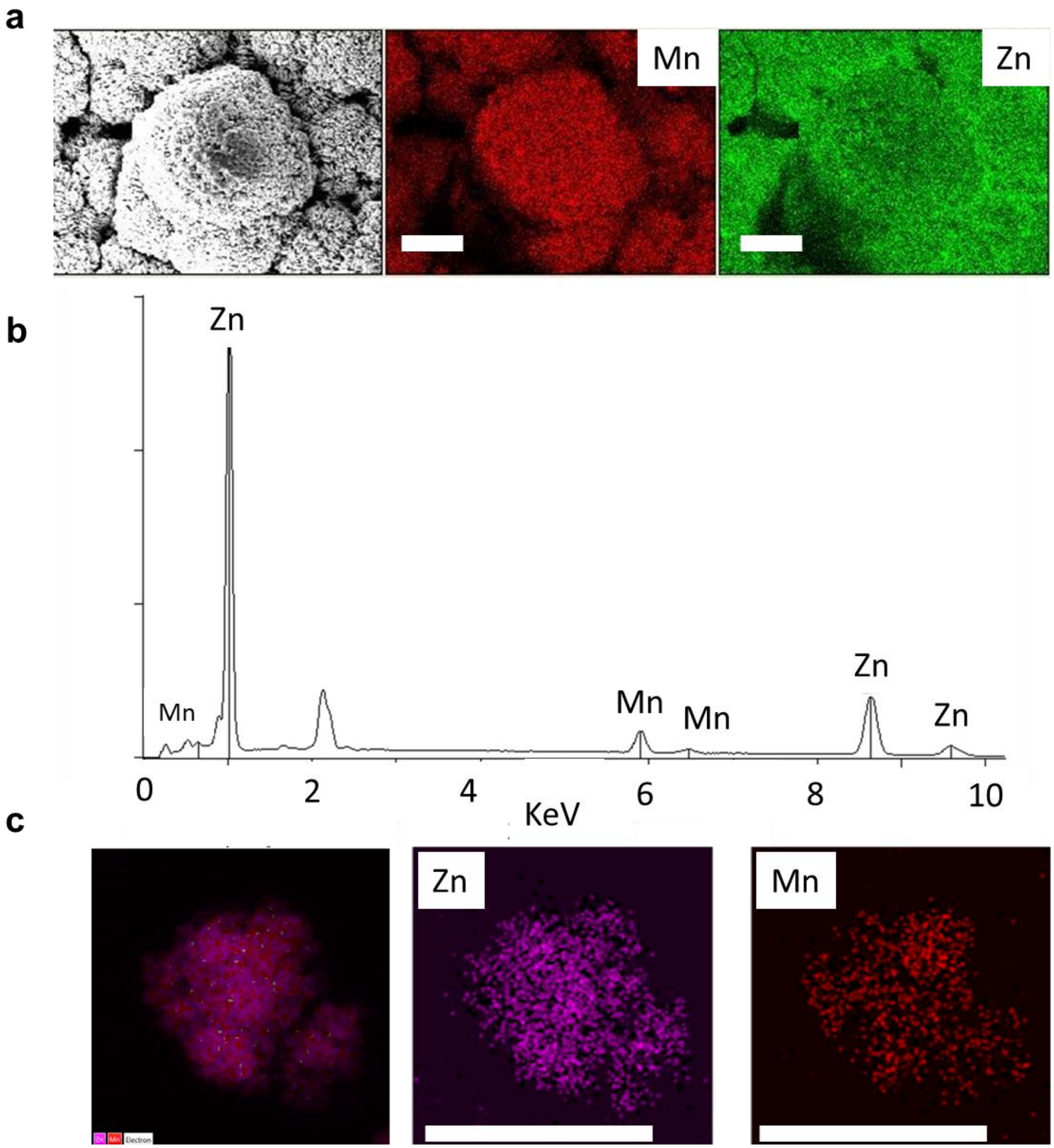

Supplementary Figure 5. Composition of Zn-Mn alloy. (a) SEM elemental mapping and (b) EDS spectra of Zn-Mn alloy prepared by 40 min deposition. Scale bars: 25 µm in (a). (c) TEM mapping of Zn-Mn alloy. Scale bars: 50 nm in (c). The elemental mapping and EDS spectra confirm the alloy composition.

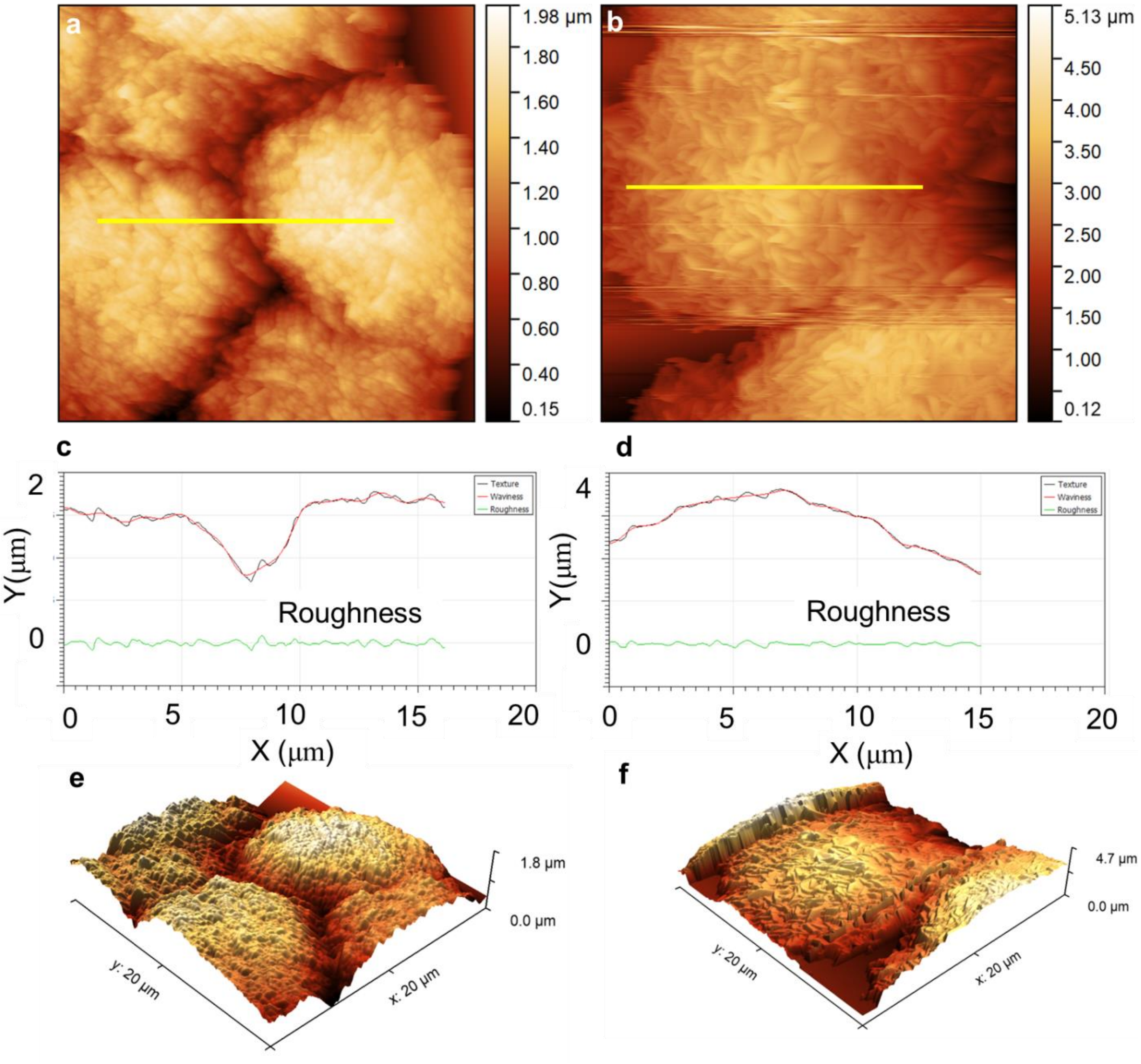

Supplementary Figure 6. AFM topographies of Zn-Mn alloy. (a) Before and (b) after Zn plating at a current density of 0.5 mA cm^{-2} (Areal capacity: 5.0 mAh cm^{-2}). The corresponding line profiles were plotted in (c) and (d), respectively. 3D AFM images of (e) before and (f) after Zn plating.

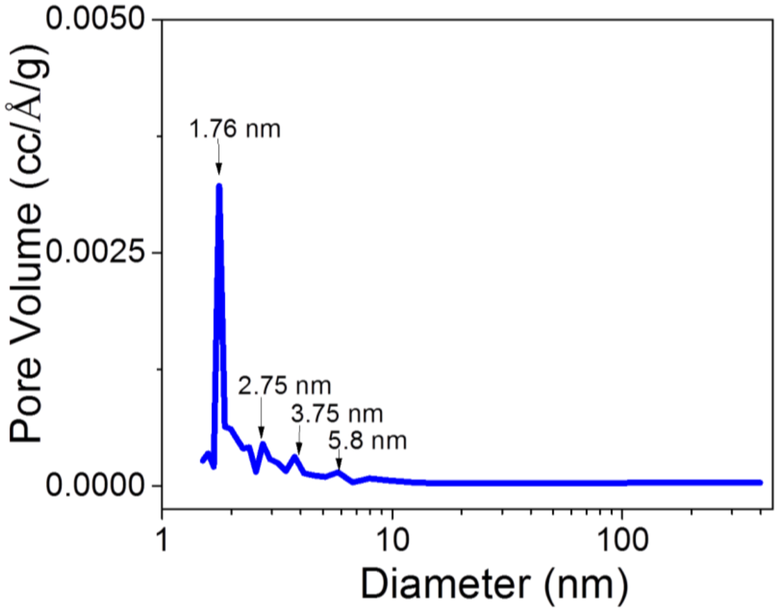

Supplementary Figure 7. Brunauer–Emmett–Teller (BET) pore size distributions of Zn-Mn alloy.

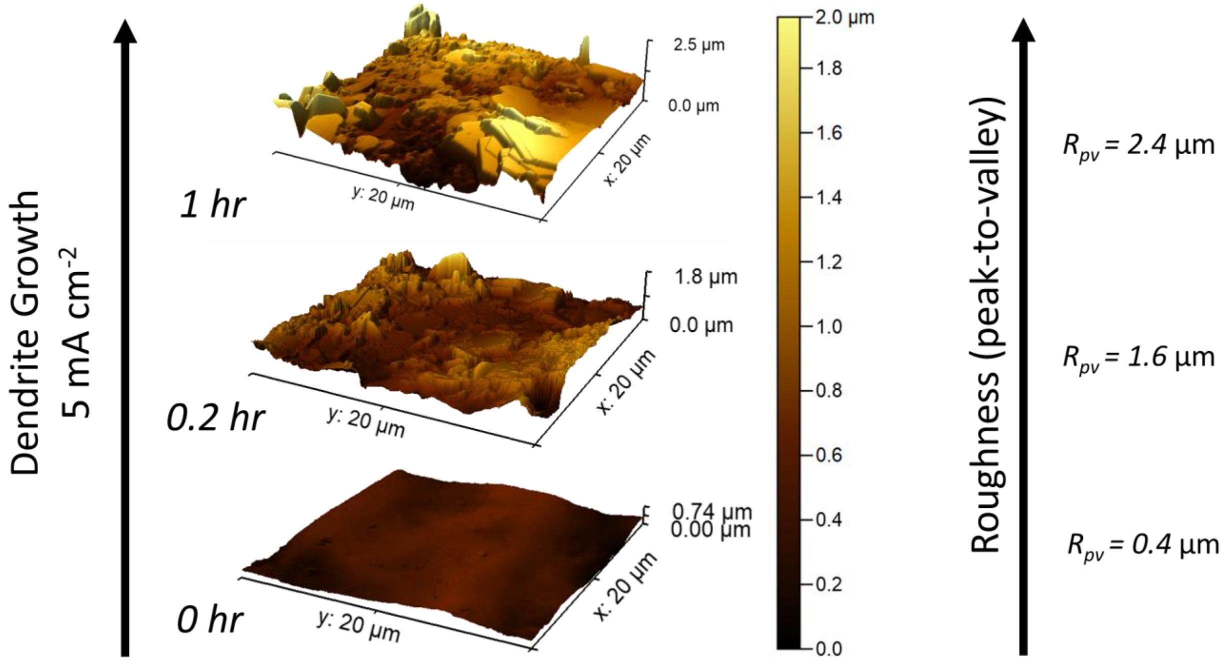

Supplementary Figure 8. AFM topographies of Zn before and after Zn plating at a current density of 5.0 mA cm^{-2}.

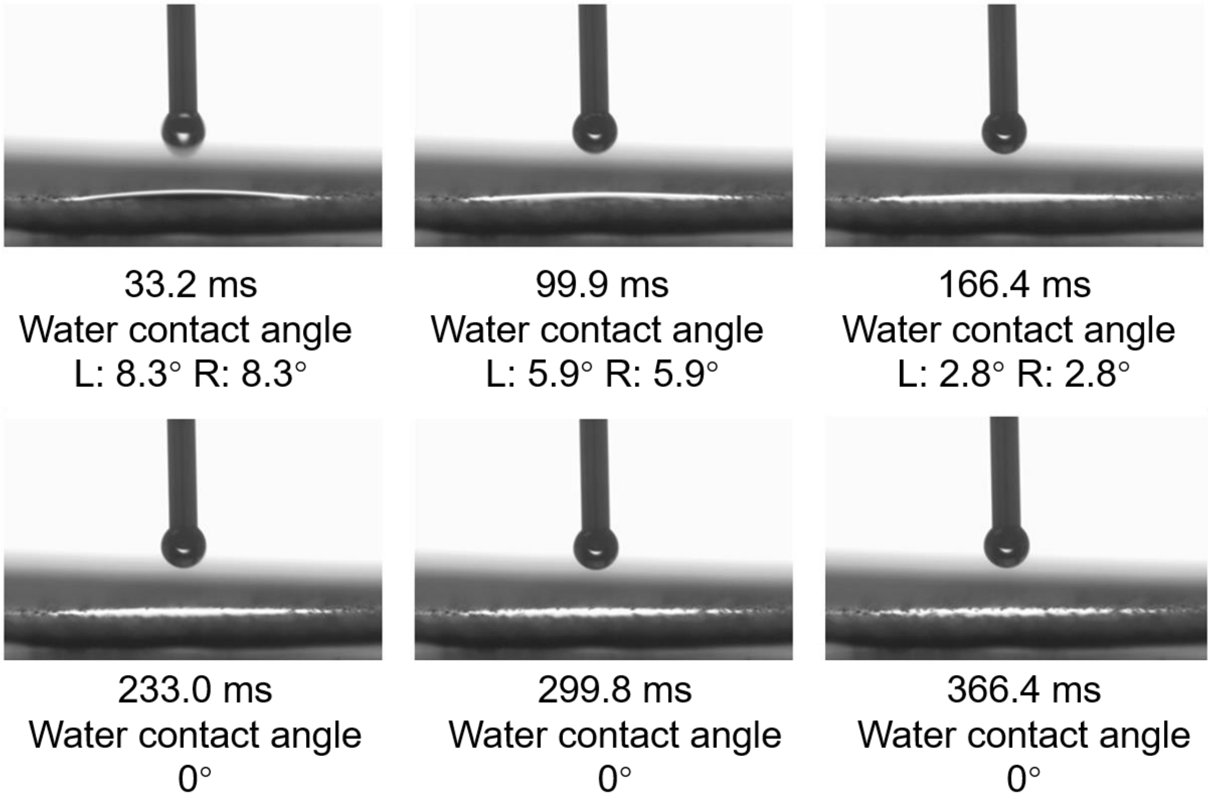

Supplementary Figure 9. Dynamic contact angle measurements on the Zn-Mn alloy. Just after 233ms, a superhydrophilicity was observed with an instantaneously wetted surface by the water droplet until the water was evaporated from the surface.

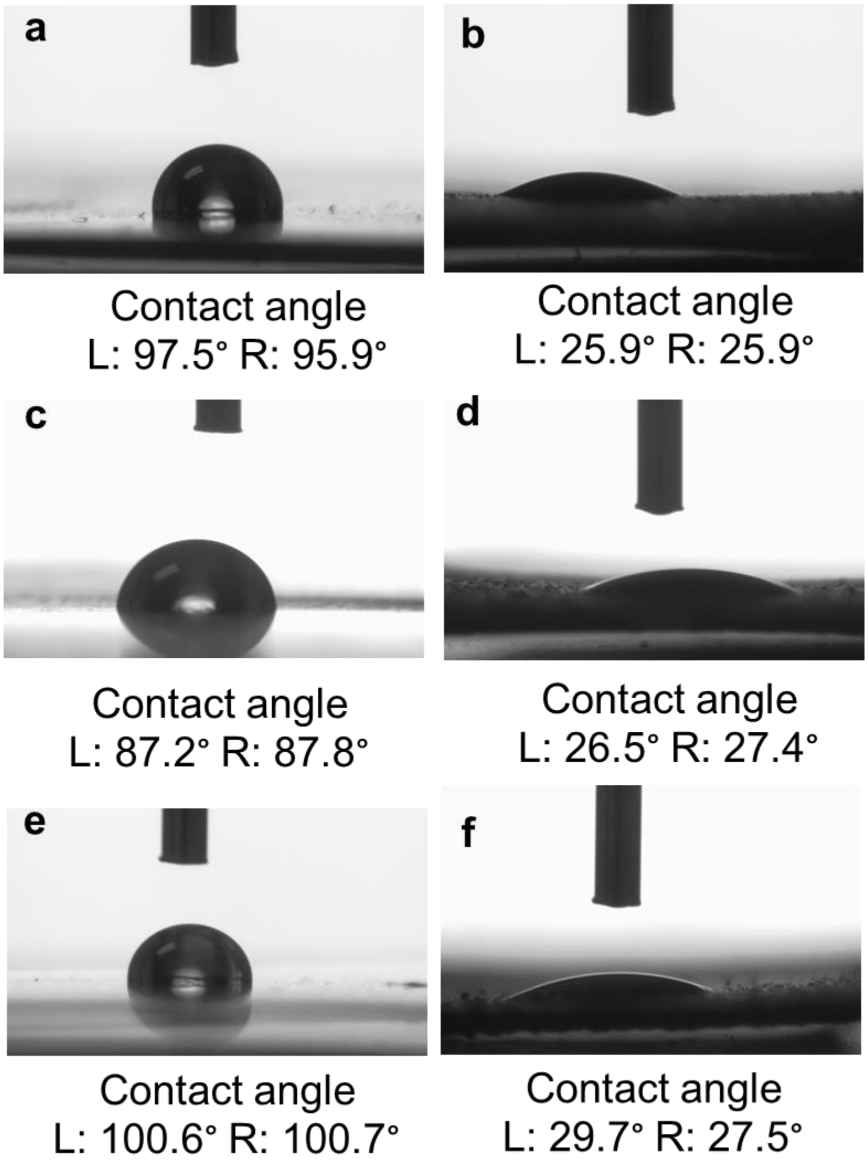

Supplementary Figure 10. Contact angle measurements. (a,c,e) The pristine Zn and (b,d,f) Zn-Mn alloy under different electrolytes. Electrolyte 1: 2 M $ZnSO_4$ with 0.1 M $MnSO_4$ in DI water for (a, b); Electrolyte 2: 2 M $ZnSO_4$ in DI water for (c, d); Electrolyte 3: 2 M $ZnSO_4$ and 0.1 M $MnSO_4$ in seawater for (e, f).

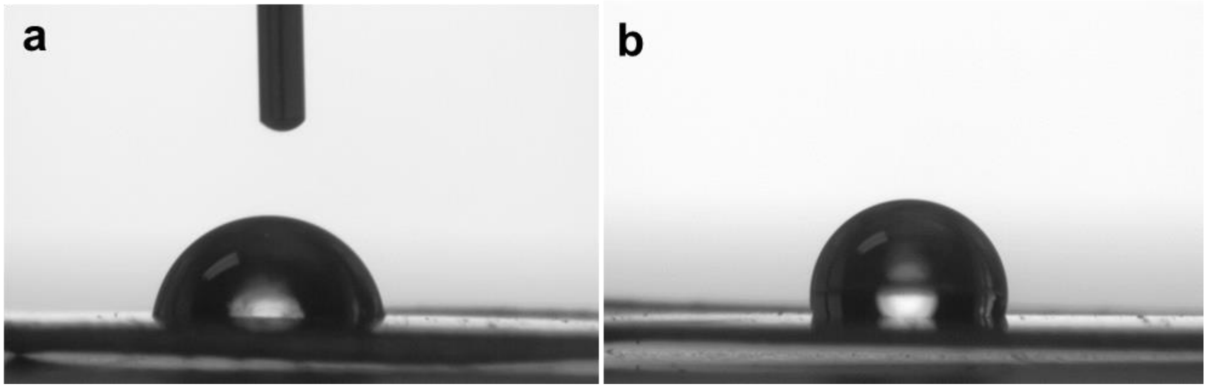

Supplementary Figure 11. Contact angle measurements. (a) Zn foil with the scratched surface (CA: L: 82.2°; R: 82.2°). (b) The pristine Zn foil (CA: L: 102.6°; R: 82.2°). Compared with the pristine Zn foil, the Zn foil with scratches had a better wettability due to the higher surface roughness.

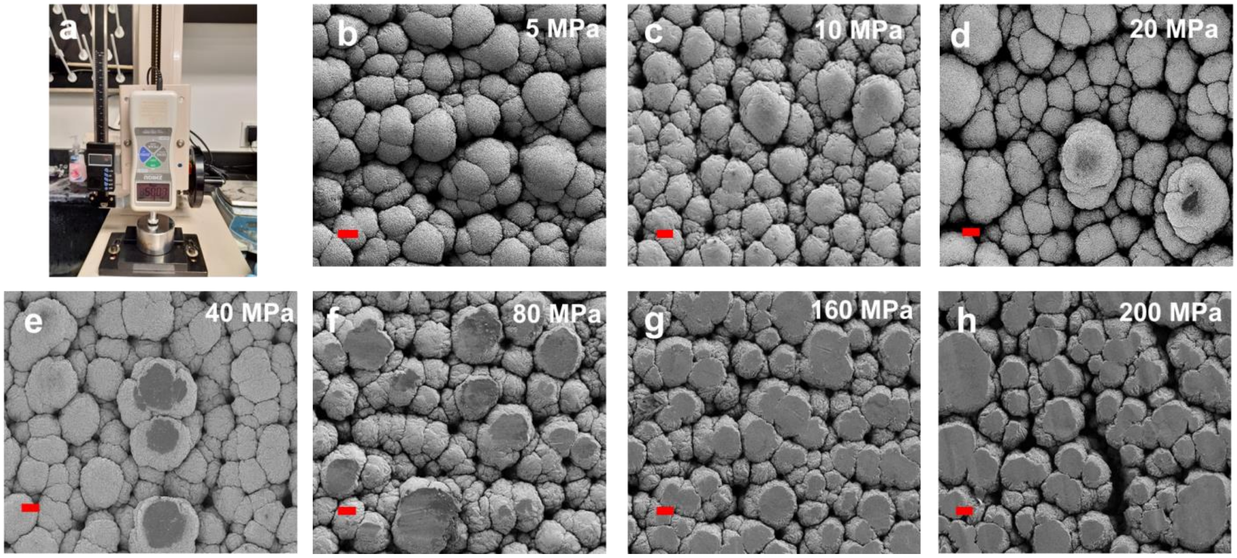

Supplementary Figure 12. Measurements of the Zn-Mn alloy after the calendering process. (a) Optical image of the calendering instrument and (b-h) SEM images of the Zn-Mn alloy after a calendering process under a different pressure in a range from 5 to 200 MPa. Scale bars: 20 μm.

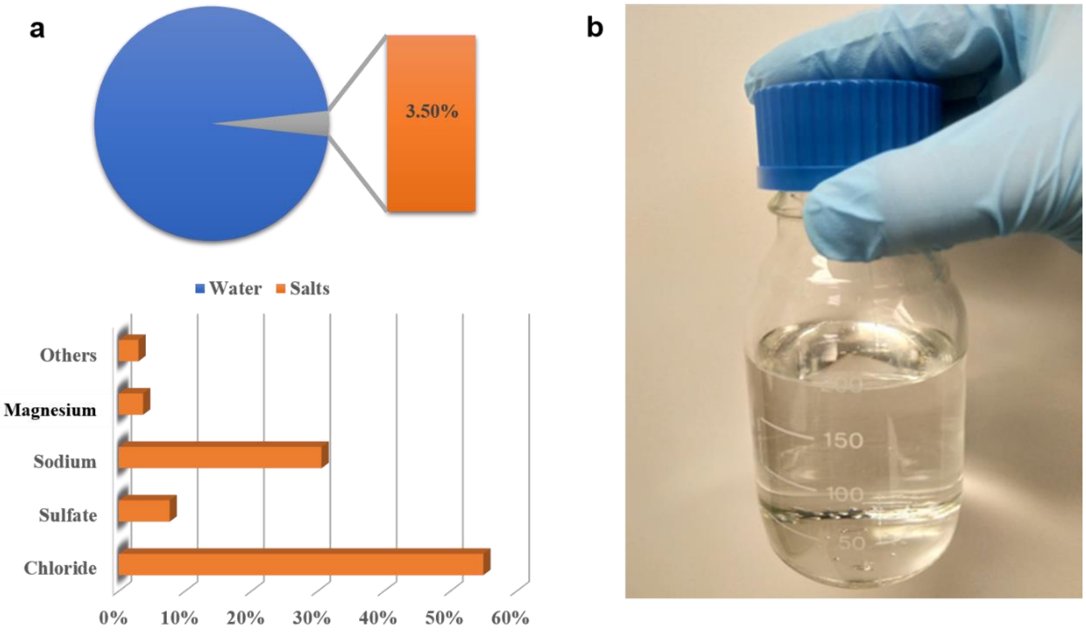

Supplementary Figure 13. Seawater-based aqueous electrolytes. (a) The components of typical seawater, including 96.5% water and 3.5% salts. (b) Photograph of seawater-based aqueous electrolyte (2 M $ZnSO_4$ in seawater). The seawater used in this work was only physically filtered to remove the suspended particles without any other treatment.

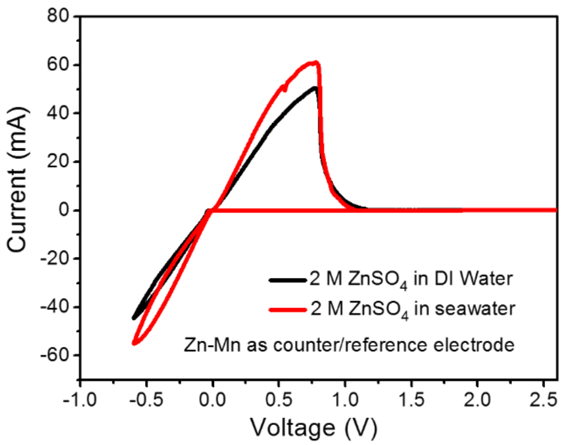

Supplementary Figure 14. Electrochemical performance of Zn-Mn alloy in aqueous electrolytes. CV curves of seawater and DI water-based electrolytes. Scan rate: 1 mV s^{-1}. Working electrode: Pt. Reference and counter electrodes: Zn-Mn alloy.

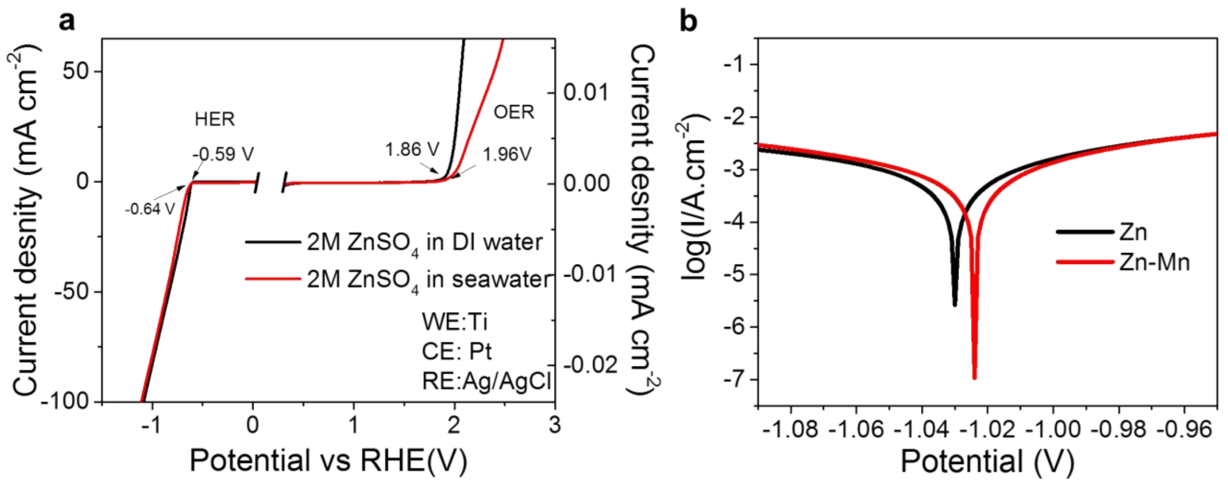

Supplementary Figure 15. Characterizations of the electrochemical stability window and anti-corrosion ability. (a) The linear sweep voltammetry curves of the DI water and seawater based aqueous electrolytes at 5 mV s^{-1}, where the potentials were referred to as a reversible hydrogen electrode (RHE). (b) Tafel curves of Zn and Zn-Mn alloy in seawater.

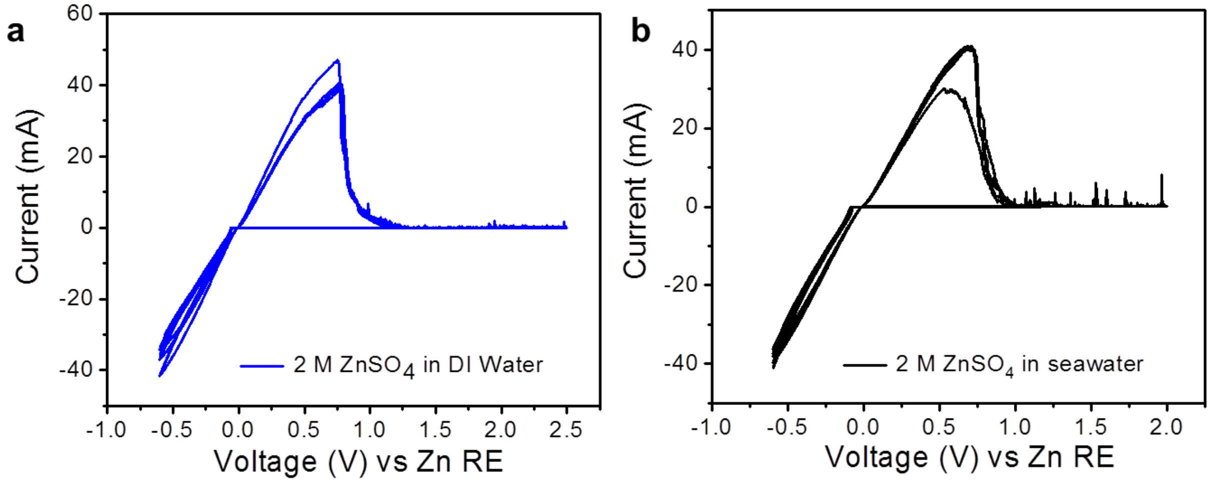

Supplementary Figure 16. Electrochemical performance of pristine Zn in aqueous electrolytes. CV test in different electrolytes (Electrolyte A: 2 M $ZnSO_4$ in DI water; Electrolyte B: 2 M $ZnSO_4$ in seawater), illustrating the electrochemical stability under Zn plating/stripping processes. Electrolyte A had a little better stability than Electrolyte B, confirming the corrosion at the interface of seawater/Zn. Working electrode: Pt. Reference and counter electrode: pristine Zn.

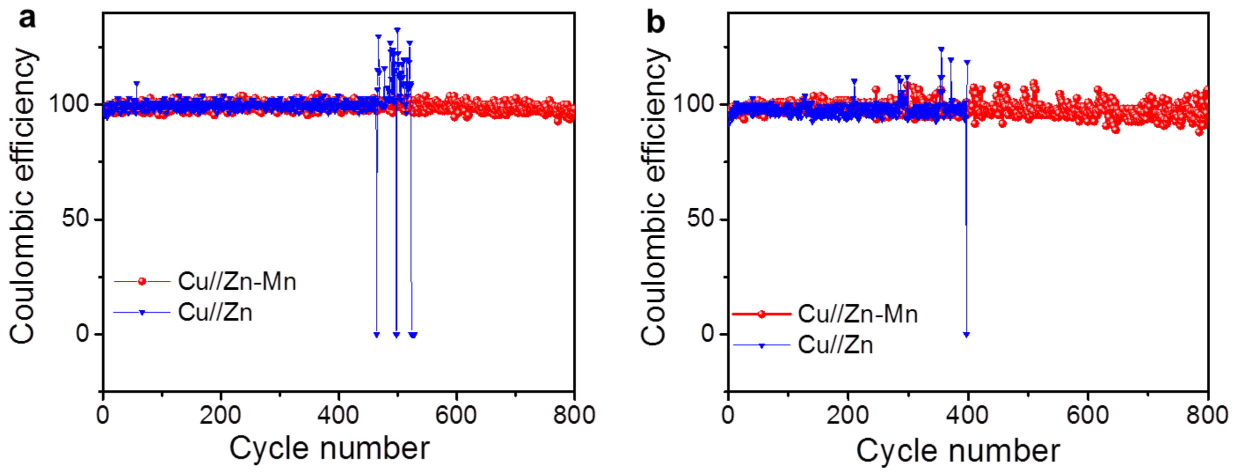

Supplementary Figure 17. Electrochemical performance of Cu//Zn and Cu//Zn-Mn coin cells using seawater-based electrolytes. Coulombic efficiencies of the Zn plating/stripping at current densities of (a) 20 mA cm^{-2} and (b) 30 mA cm^{-2}. Working electrode: Cu. Counter electrode: Zn foil or Zn-Mn alloy. The electrolyte: 2 M $ZnSO_4$ and 0.1 M $MnSO_4$ in seawater. At high current densities of 20 mA cm^{-2} and 30 mA cm^{-2}, the Cu//Zn cells were short-circuited after 450 cycles due to the dendrite growth. The Cu//Zn-Mn cells maintained very stable performance even after 800 cycles, indicating the superior stability in the seawater-based electrolyte.

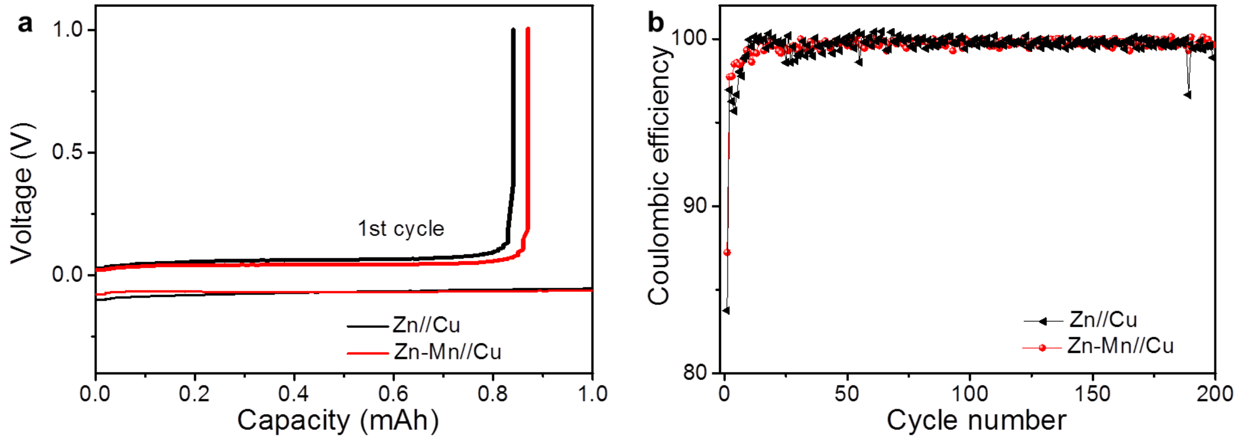

Supplementary Figure 18. Electrochemical performance of Cu//Zn and Zn-Mn//Cu coin cells using DI water-based electrolytes. (a) Charge-discharge voltage gap in the 1st cycle and (b) Coulombic efficiency (CE) stability of Zn and Zn-Mn alloy at a current density of 5 mA cm^{-2} (areal capacity: 1.0 mAh cm^{-2}) using Cu as cathodes in the electrolyte consisting of 2 M $ZnSO_4$ in water. At a current density of 5 mA cm^{-2}, the Zn-Mn//Cu cells present a higher initial CE with a lower polarization of 109 mV (137 mV for Zn//Cu cells) and keep stable cycling performance for over 200 cycles.

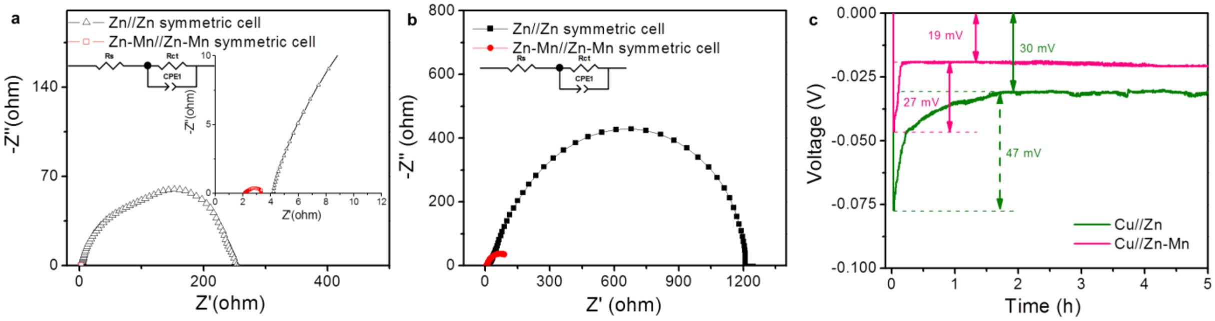

Supplementary Figure 19. Electrochemical impedance spectroscopy (EIS) of the symmetric Zn//Zn and Zn-Mn//Zn-Mn cells. (a) Seawater-based electrolyte (2 M $ZnSO_4$ in seawater) and (b) DI water-based electrolyte (2 M $ZnSO_4$ in DI water). (c) Nucleation overpotentials of Zn-Mn alloy and pristine Zn asymmetric cells (vs. Cu electrode) at a current density of 1.0 mA cm^{-2}.

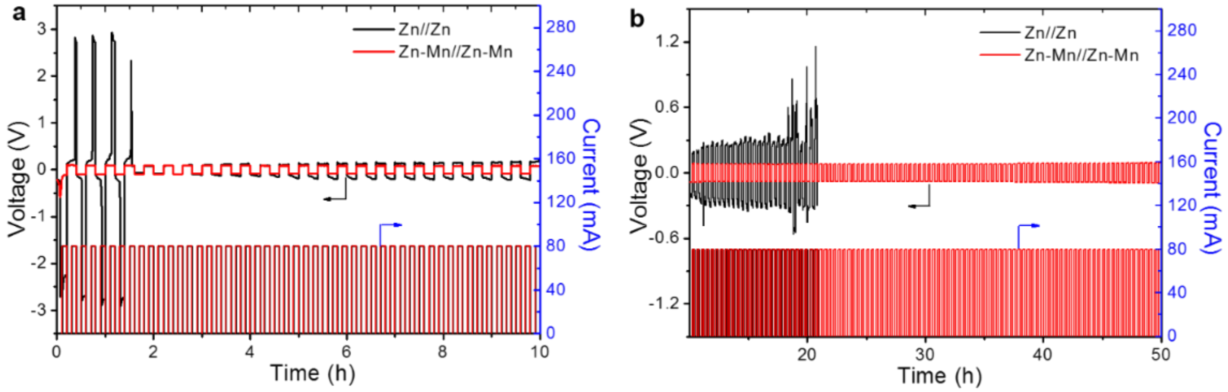

Supplementary Figure 20. Plating/stripping curves of the symmetric Zn-Mn//Zn-Mn and Zn//Zn cells. At a current density of 80 mA cm^{-2} (areal capacity: 16 mAh cm^{-2}) in the range of (a) 0-10 h and (b)10-50 h. Electrolyte: 2 M $ZnSO_4$ in seawater.

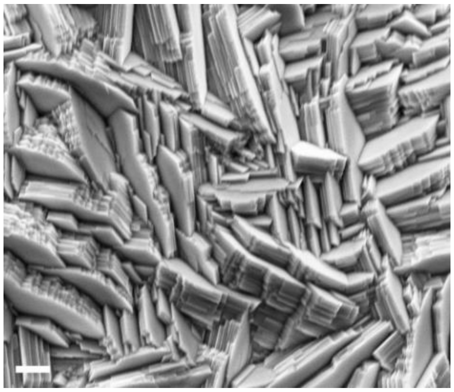

Supplementary Figure 21. The morphology of Zn@Zn anode. SEM image of Zn@Zn foil anode. Scale bar: 1 μm.

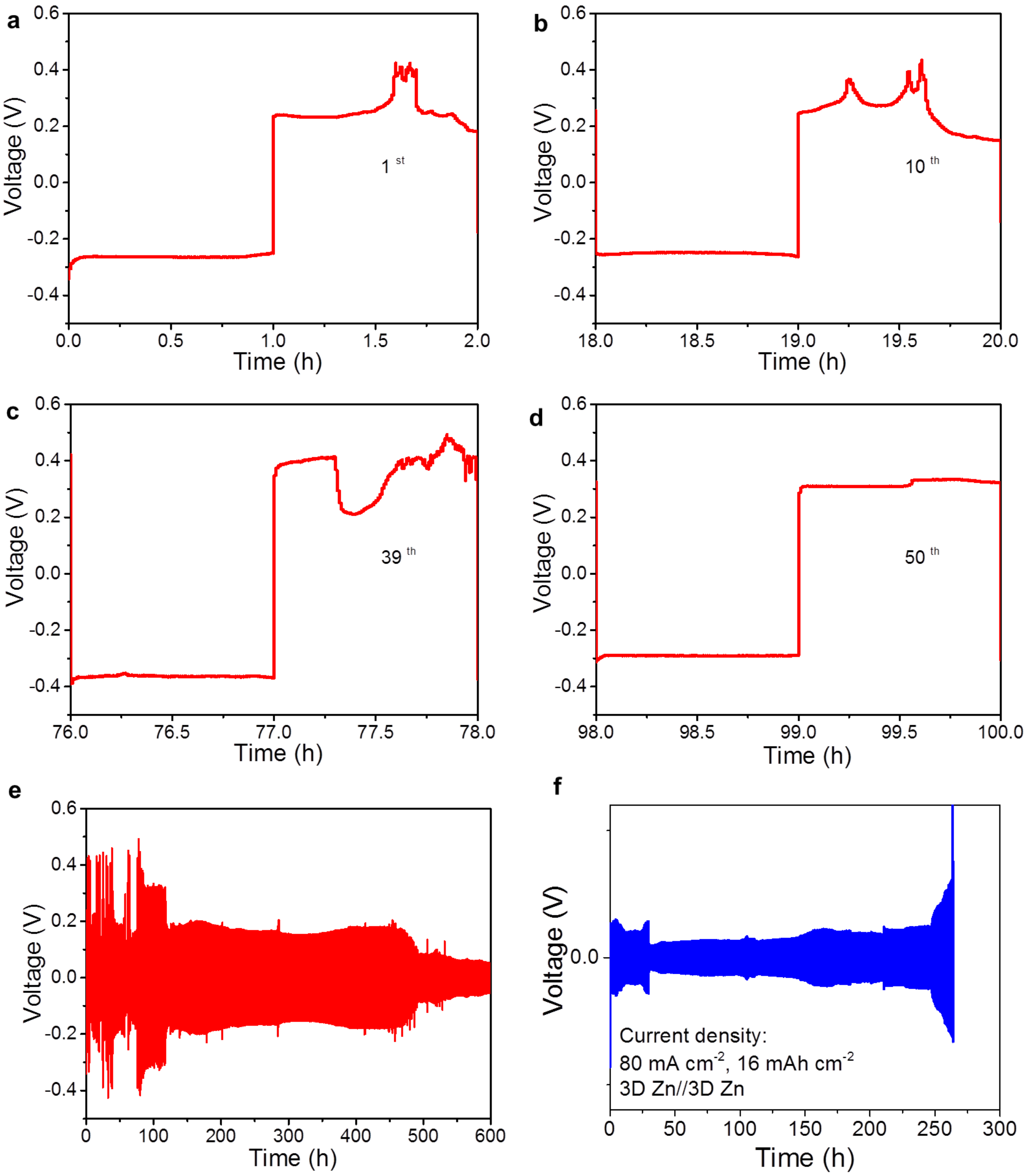

Supplementary Figure 22. The plating/stripping profiles of symmetric Zn@Zn//Zn@Zn cells. (a) 1st, (b) 10th, (c) 39th, and (d) 50th cycle at a current density of 5 mA cm^{-2}. (e) and (f) Long-term cycling performance at a current density of 5 mA cm^{-2} (areal capacity: 5.0 mAh cm^{-2}) and 80 mA cm^{-2} (areal capacity: 16 mAh cm^{-2}), respectively. Electrolyte: 2 M $ZnSO_4$ in seawater.

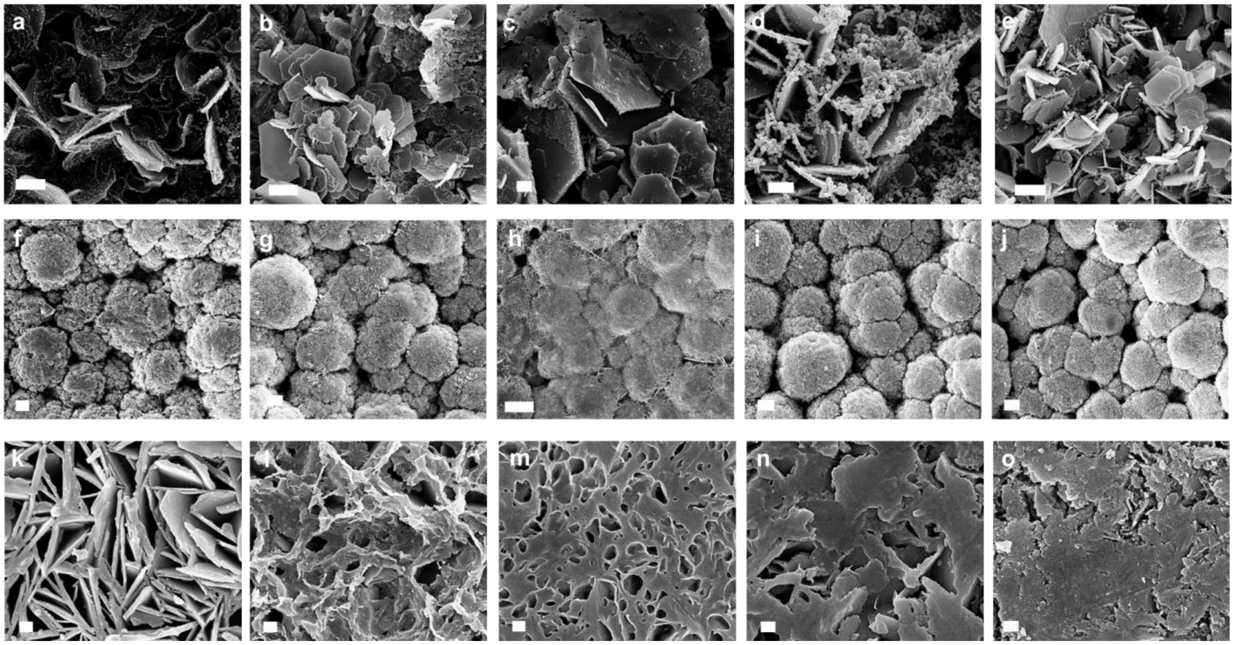

Supplementary Figure 23. Morphologies of Zn and Zn-Mn alloy after Zn plating at different current densities of 1, 5, 10, 50, and 80 mA cm^{-2} (from left columns to right columns, areal capacity: 1.0 mAh cm^{-2}). (a-e) SEM images of Zn. Scale bars: 1 µm. (f-j) SEM images of Zn-Mn alloy. Scale bars: 10 µm. (k-o) High-magnification SEM images of Zn-Mn alloy. Scale bars: 200 nm.

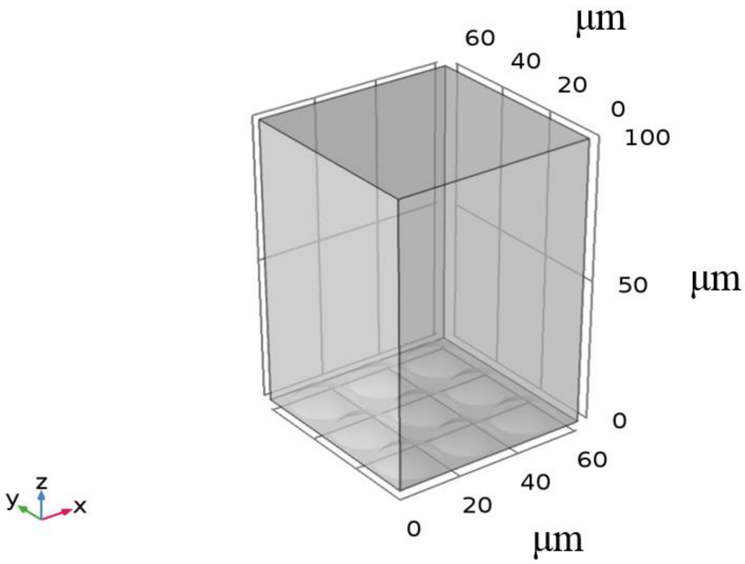

Supplementary Figure 24. The geometry of the 3D COMSOL model. The half-spheres at the bottom were used to mimic the 3D Zn-Mn alloy structures.

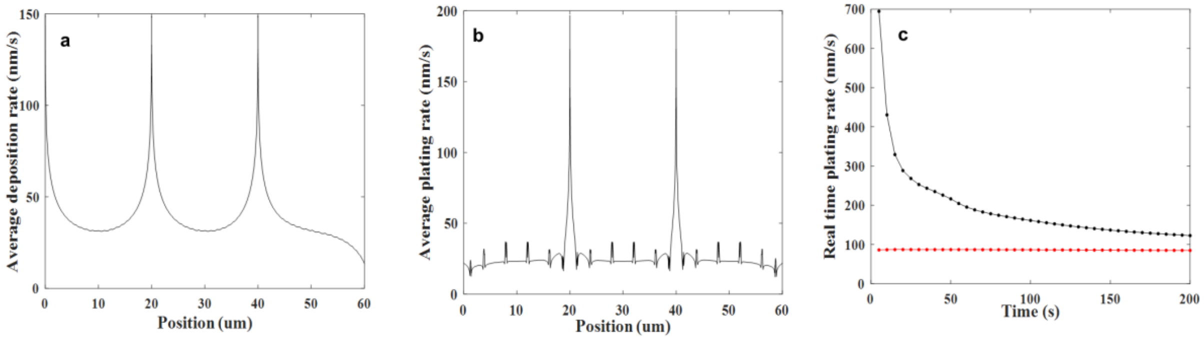

Supplementary Figure 25. 2D COMSOL model to simulate the Zn plating rate. (a) Average plating rate from **Model 2** after 30s plating. (b) Average plating rate from **Model 3** after 10s plating. (c) Plating rate from **Model 1** in the first 200s. The black and red lines represent Zn plating rates in the deepest trench and on the highest protrude area, respectively.

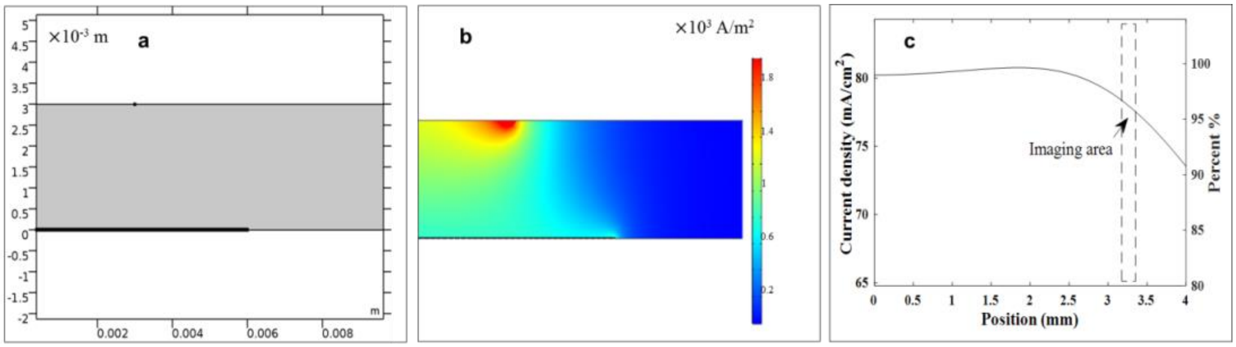

Supplementary Figure 26. 2D COMSOL Model 1. (a) Geometry model. (b) Current density distribution. (c) Current density distribution across the bottom electrode. The dashed lines show the imaging location in the experiments. The current density at the imaging area is 95% of the maximum values between two electrodes.

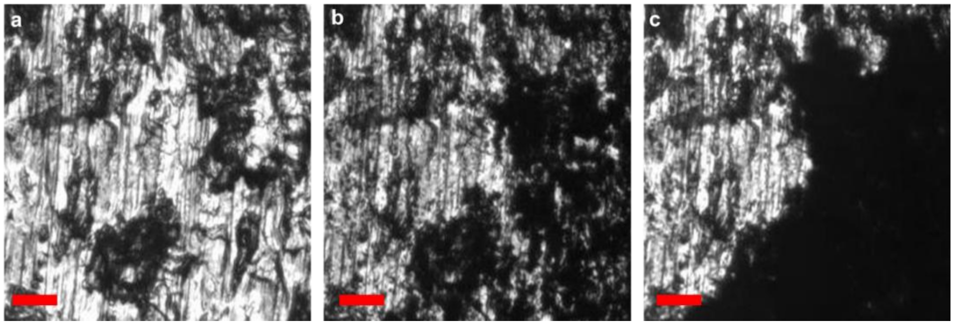

Supplementary Figure 27. Dendrite growth on the pristine Zn surface imaged by the *in-situ* optical microscope. The Zn surface was cleaned before the experiment. Images were taken with a 20X water immersion objective at 26 frames per second and the experiment was performed at a current density of 30 mA cm^{-2}. (a) Zn foil before plating. (b) Zn plating after 60s. (c) Zn plating after 720s. Scale bars: 15 µm.

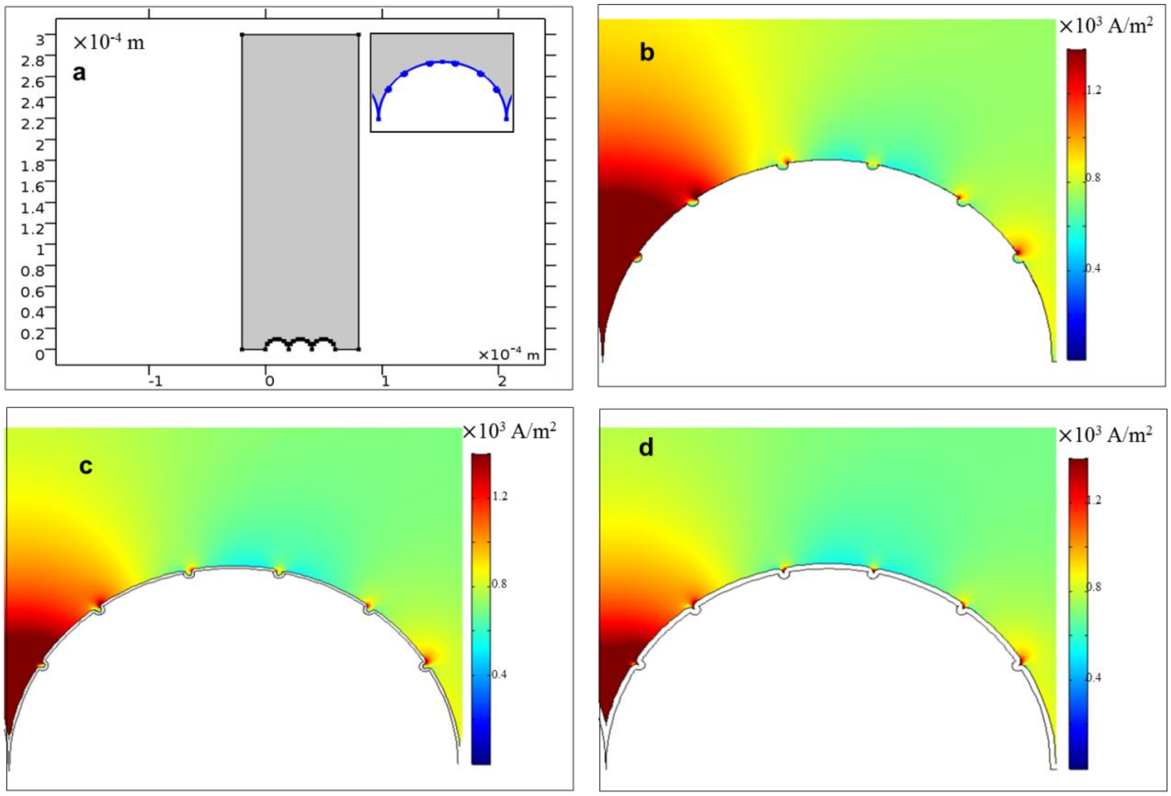

Supplementary Figure 28. 2D COMSOL Model 2 with nano-voids. (a) Geometry model. (b) Current density distribution and the bottom electrode profile. (c) Current density distribution and electrode profile after 5s of plating. (d) Current density distribution and electrode profile after 10s of plating.

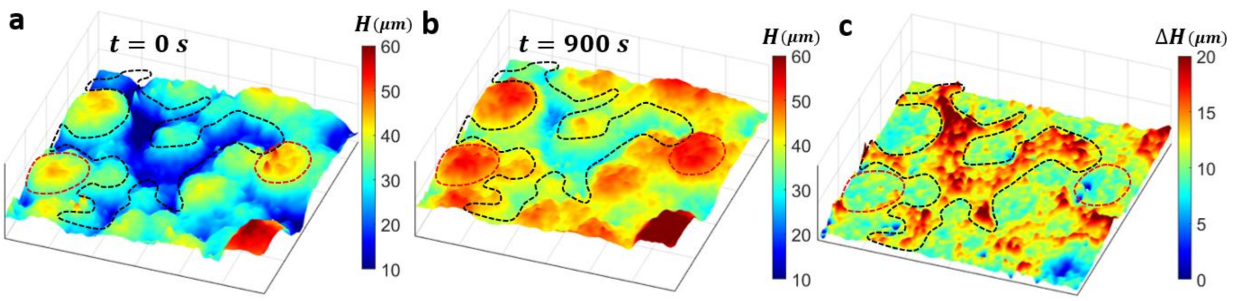

Supplementary Figure 29. 3D morphology of the Zn-Mn electrode before and after deposition imaged by the *in-situ* optical microscopes. (**a**) Original 3D profile of the Zn-Mn electrode. (**b**) 3D profile after 900s of Zn deposition with 80 mA cm^{-2} current density. (**c**) The thickness change caused by the Zn deposition for 900s. From figure (c) we can see that the Zn platting is much faster in the trench regions (the regions circled by the black dashed line) than that on the protruding regions (the regions circled by the red dashed lines). Note that the colormap here represents the real morphology. This result further verifies our observation in **Fig. 3**.

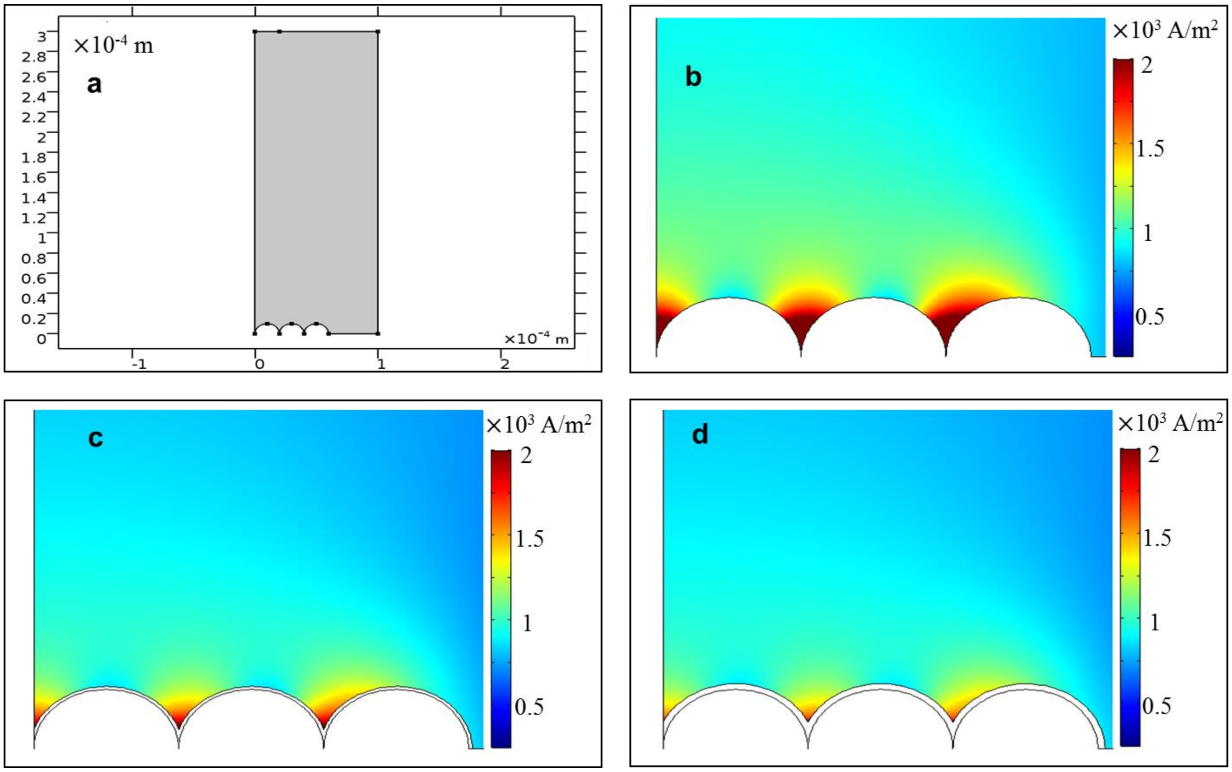

Supplementary Figure 30. 2D COMSOL Model 3. (a) Geometry model. (b) Current density distribution and the original electrode profile. (c) Current density distribution and electrode profile after 15s of plating. (d) Current density distribution and electrode profile after 30s of plating.

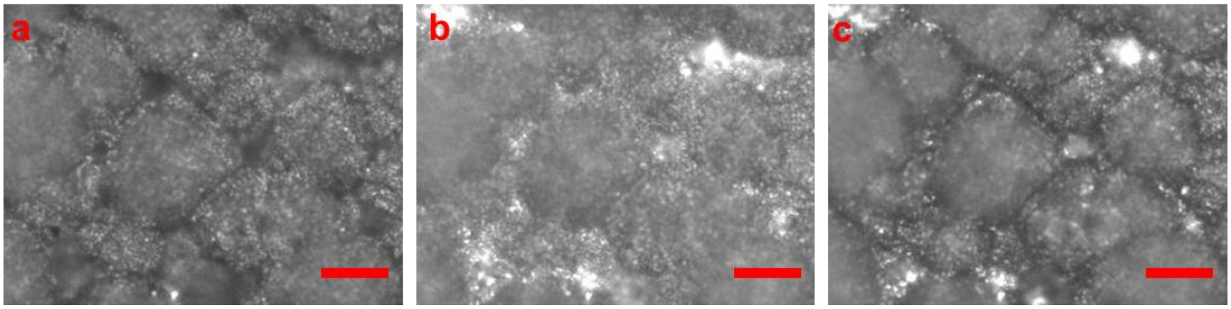

Supplementary Figure 31. *In-situ* imaging of the Zn plating/stripping process. 3D Zn-Mn surface (a) before plating, (b) after 160s plating, (c) after 240s stripping. Scale bars: 15 µm.

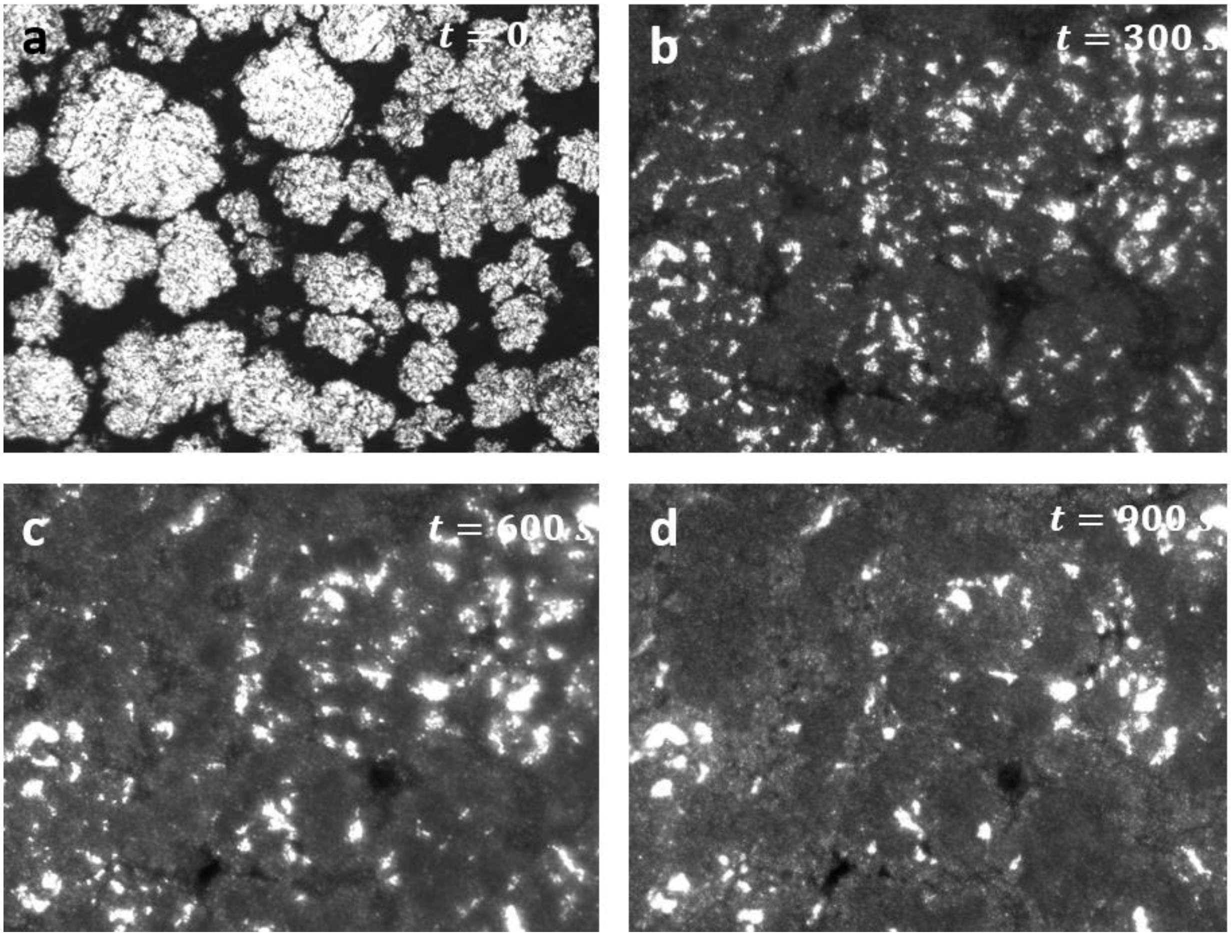

Supplementary Figure 32. Plane Zn_3Mn test. (**a**) Flattened Zn_3Mn surface. The surface is compressed with the mechanical force. The bright region is the flat region which covers most of the region. The dark regions are the original trench regions. (**b-d**) The images after 300s, 600s, and 900s of plating under 80 mA cm^{-2}.

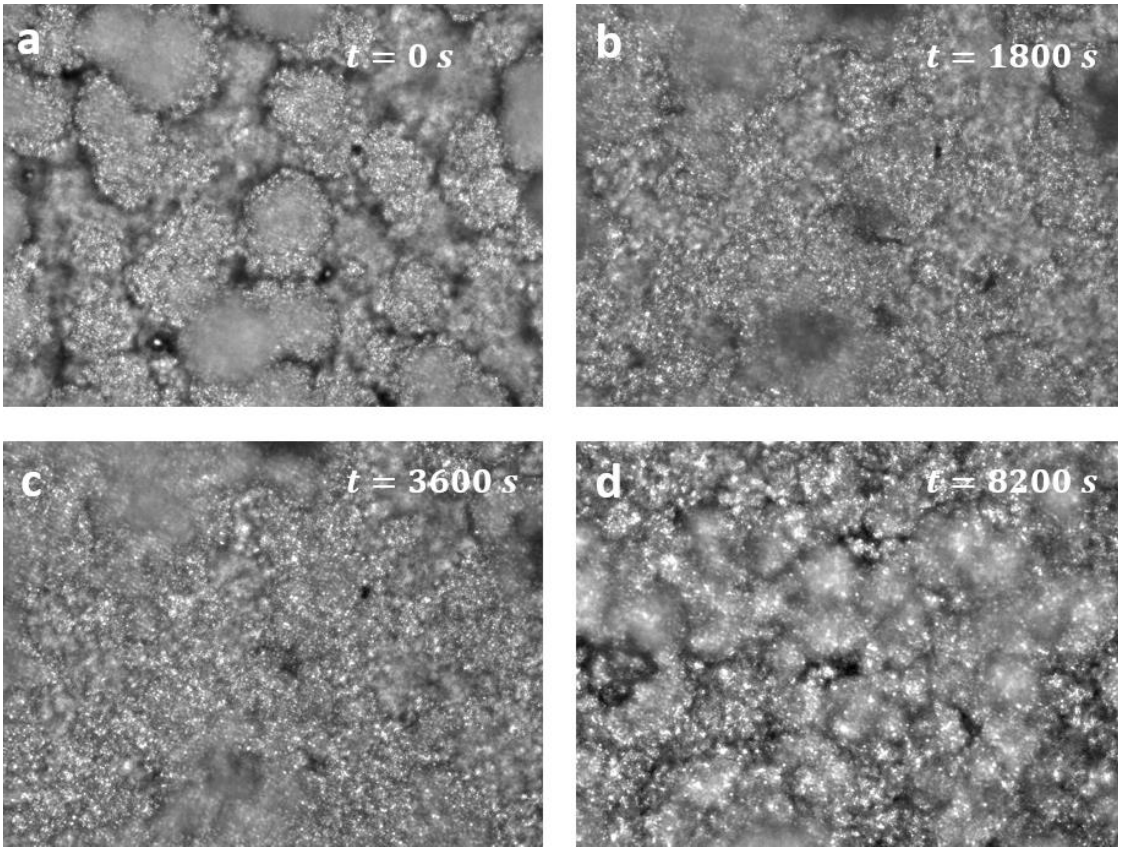

Supplementary Figure 33. The ultimate dendrite suppression capability test of 3D Zn-Mn alloy: continuous Zn plating without obvious dendrite formation. (**a-d**) Optical images of 3D Zn-Mn alloy at 0s, 1800s, 3600s, and 8200s at the current density of 80 mA cm^{-2}.

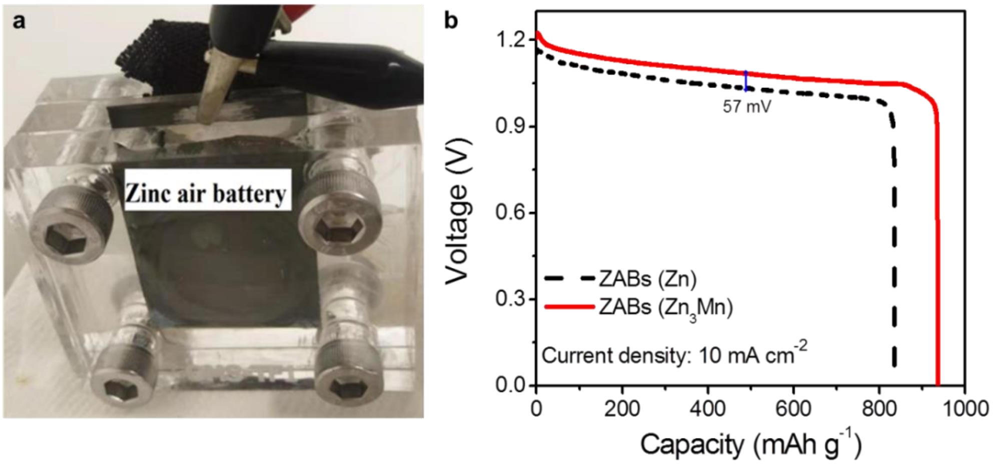

Supplementary Figure 34. Zn-air batteries (ZABs) using different metal anodes. (a) The home-made ZABs cell and (b) long-term discharging profiles of ZAB using Pt/C@RuO_2 cathode and Zn-Mn alloy (ZABs (Zn_3Mn), red solid line) or Zn anode (ZABs (Zn), black dash line) at 10 mA cm^{-2}.

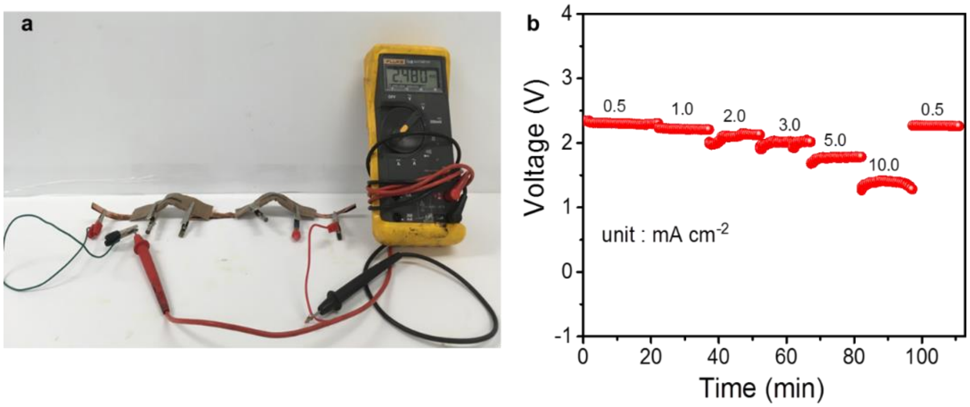

Supplementary Figure 35. Flexible ZABs. (a, b) The porotype and rate-performance of two flexible tandem ZABs, respectively.

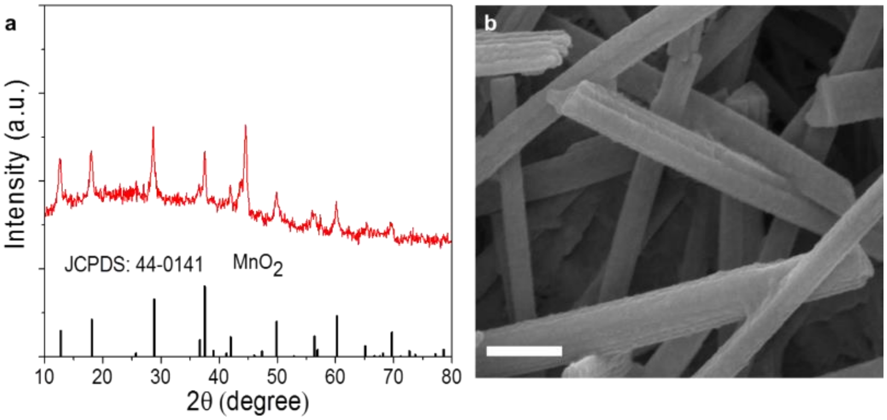

Supplementary Figure 36. Characterizations of MnO_2 cathodes for Zn-Mn//MnO_2 batteries. (a, b) XRD pattern and SEM image, respectively. Scale bar: 200 nm.

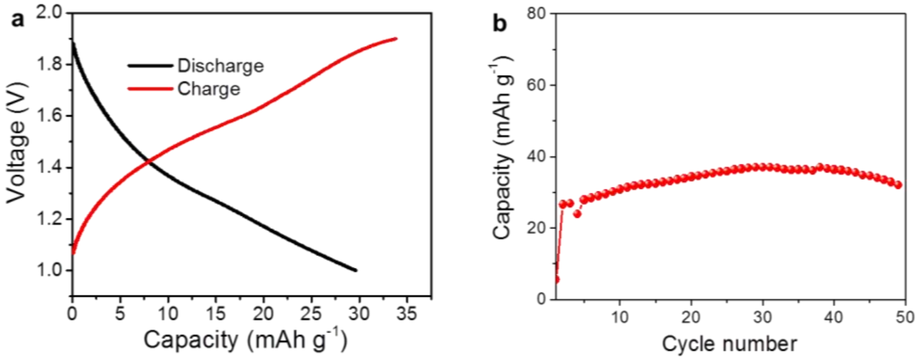

Supplementary Figure 37. Electrochemical performance of Zn-Mn//MnO_2 batteries in the seawater-based electrolyte (2 M Na_2SO_4 in seawater). (a, b) Charge/discharge profiles and cycling performance, respectively.

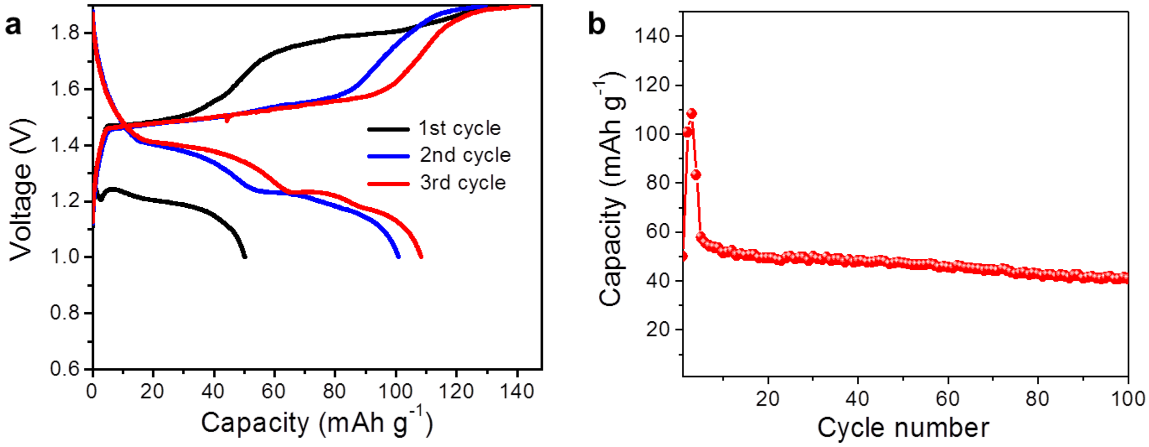

Supplementary Figure 38. Electrochemical performance of Zn-Mn//MnO_2 batteries using seawater-based electrolyte (2 M $MgSO_4$ in seawater). (a, b) Charge/discharge profiles and cycling performance, respectively.

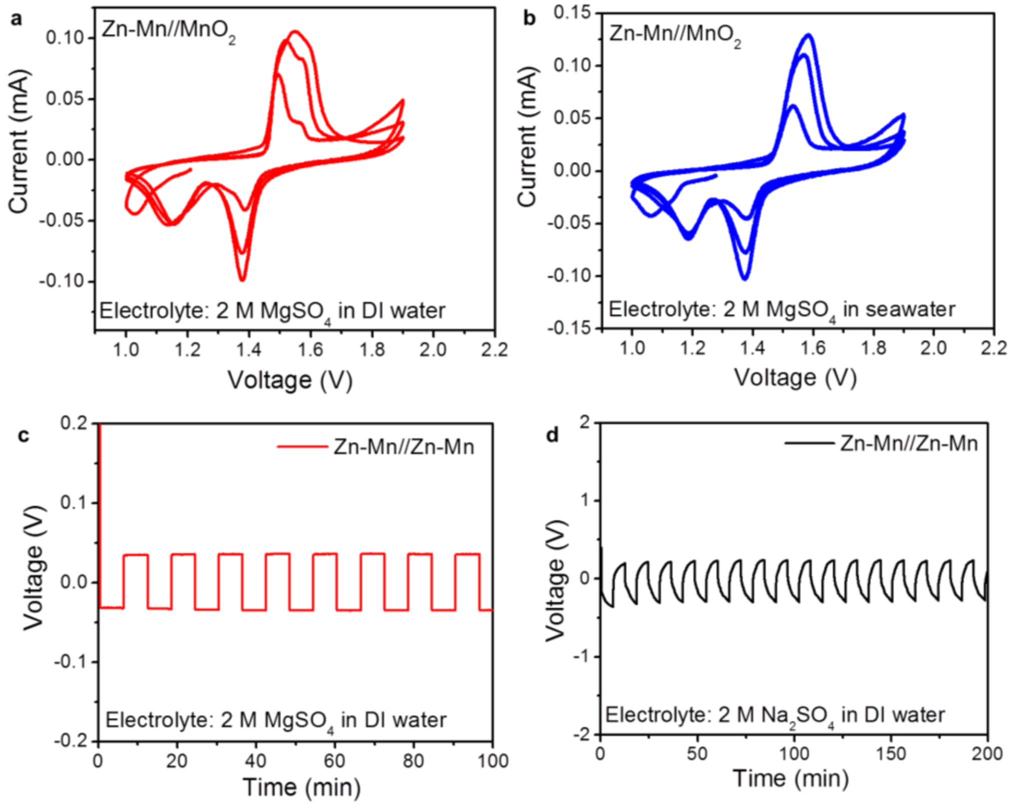

Supplementary Figure 39. The impact of hetero-ions (Na^+ and Mg^{2+}) on the electrochemical performance of Zn-Mn alloy. CV curves of Zn-Mn//MnO_2 batteries using Mg^{2+}-containing electrolytes in (a) DI water and (b) seawater. Cycling performance of the symmetric Zn-Mn//Zn-Mn cells at a current density of 1 mA cm^{-2} using (c) Mg^{2+}-containing electrolyte and (d) Na^+-containing electrolyte.

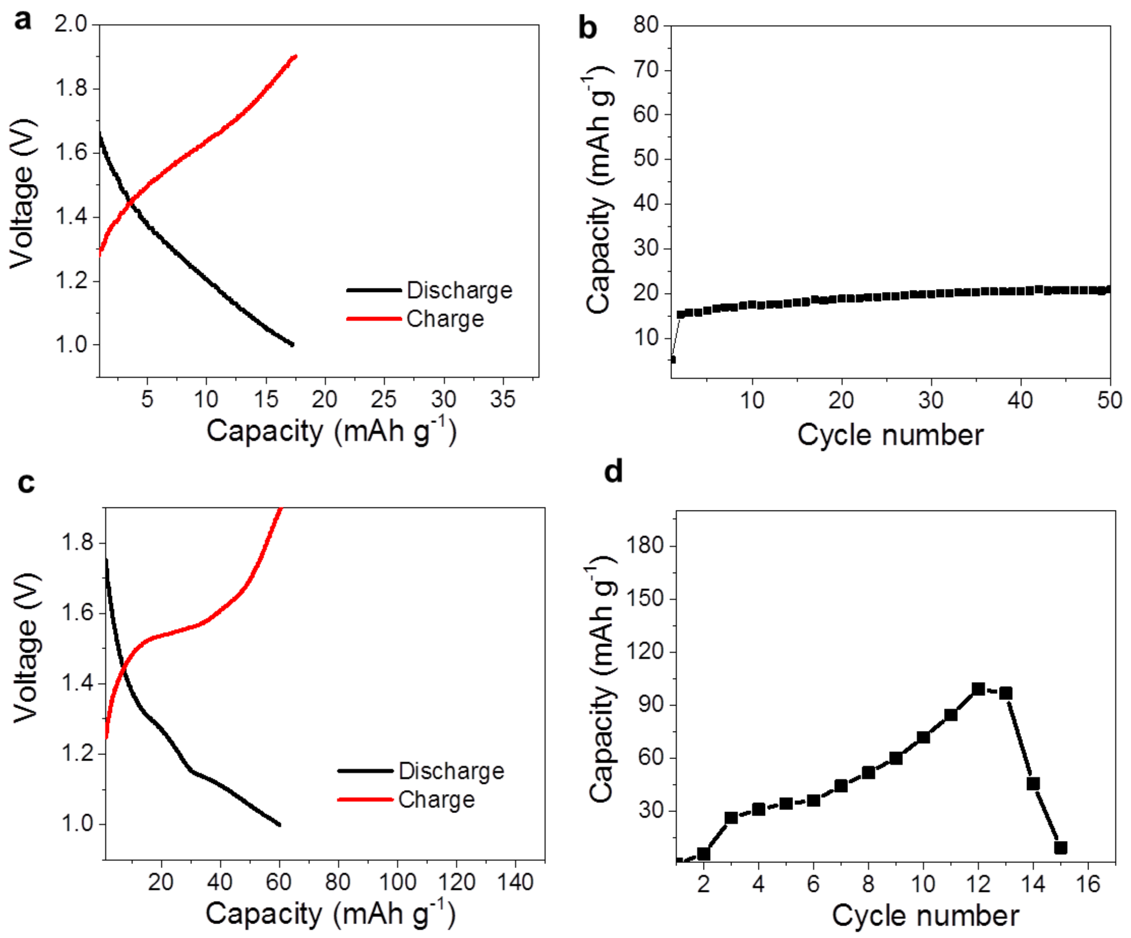

Supplementary Figure 40. Electrochemical performance of Zn//MnO_2 batteries using the seawater-based electrolyte. (a, b) Charge/discharge profiles and cycling performance, respectively (2 M Na_2SO_4 in seawater). (c, d) Charge/discharge profiles and cycling performance, respectively (2 M $MgSO_4$ in seawater).

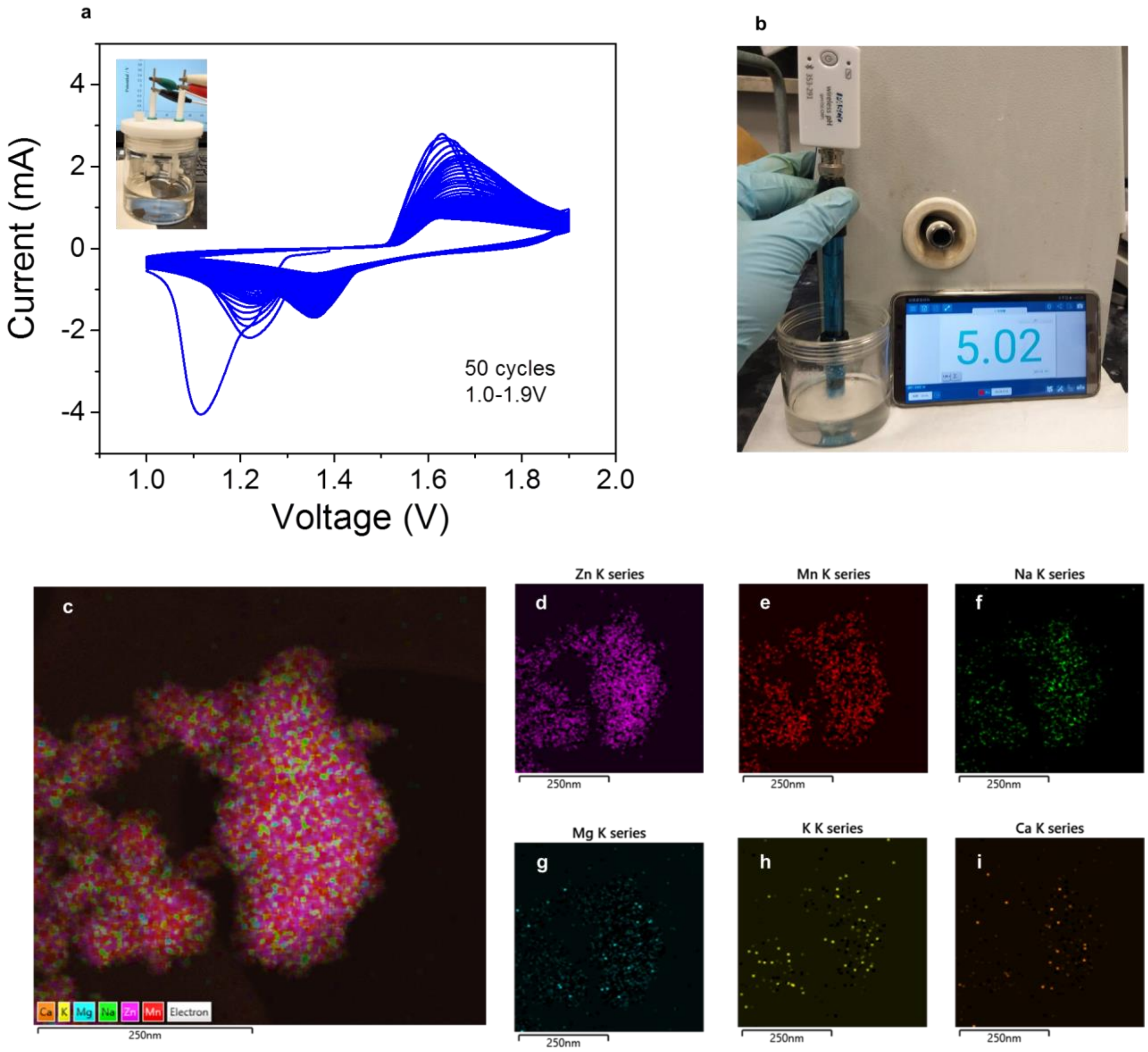

Supplementary Figure 41. The impact of hetero-ions (Cl^- and Ca^{2+}) on the electrolyte and Zn-Mn alloy. (a) Cyclic voltammetry (CV) curves of a well-sealed 2-electrode MnO_2//Zn-Mn cell. (b) The pH of the electrolyte after 50 cycles. (c) HR-TEM and (d-i) EDS images of Zn-Mn alloy after cycling in the seawater-based electrolyte (Electrolyte: 2 M $ZnSO_4$ in seawater).

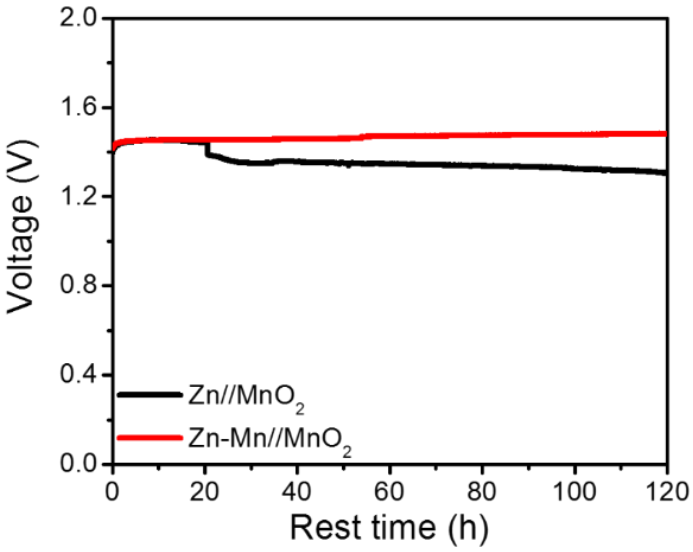

Supplementary Figure 42. Self-discharge test on Zn-ion batteries (ZIBs) using pristine Zn and Zn-Mn alloy as anodes in the seawater-based electrolyte (2 M $ZnSO_4$ in seawater). The open-circuit voltage (OCV) of ZIBs (Zn) dropped quickly after about 20h test (black line), indicating the corrosion of the pristine Zn anode. In sharp contrast, the OCV of ZIBs (Zn_3Mn) kept very stable even after 120h (5 days) without any degradation (red line), confirming the stability of Zn-Mn alloy in the seawater-based aqueous electrolyte.

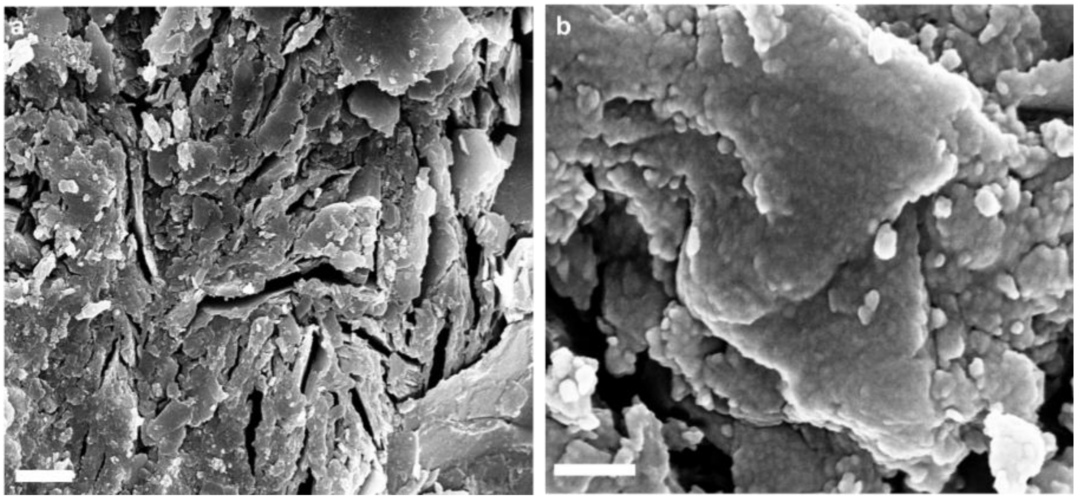

Supplementary Figure 43. Morphologies of Zn-Mn alloy after long-term cycling. (a) Low-magnification and (b) high-magnification SEM images of Zn-Mn alloy after 2000 cycles in the seawater-based electrolyte. Scale bars: 1 μm in (a) and 200 nm in (b).

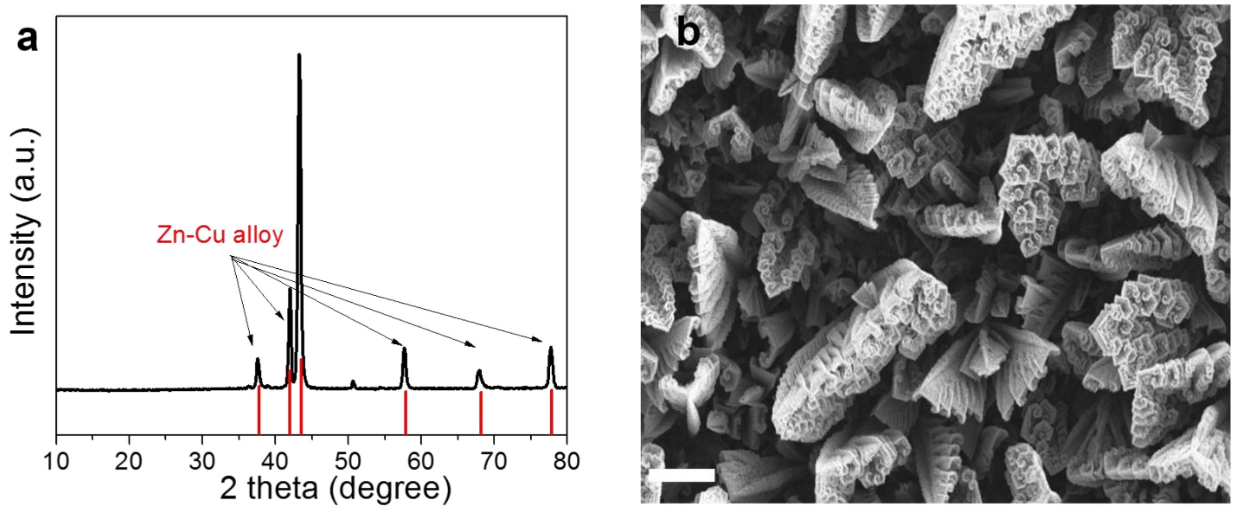

Supplementary Figure 44. Structure and morphologies of Zn-Cu alloy. (a) XRD pattern and (b) SEM image of Zn-Cu alloy. XRD pattern confirmed the Zn-Cu alloy phase. SEM image showed the 3D structure.

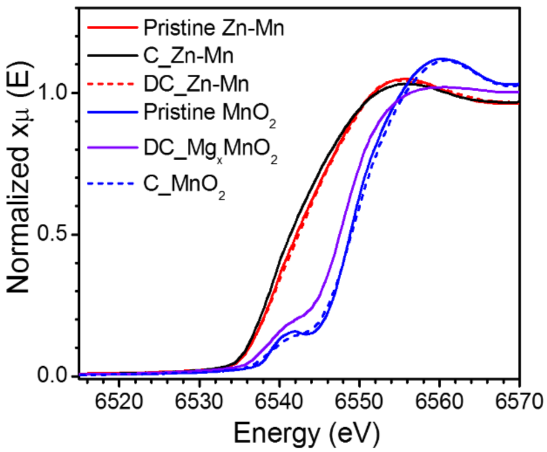

Supplementary Figure 45. XAS analysis. Mn K-edge XANES of Zn-Mn alloy at different cycling states (pristine, fully discharged (DC) and fully charged (C) Zn-Mn) and MnO_2 at different cycling states (pristine, DC, and C_MnO_2).

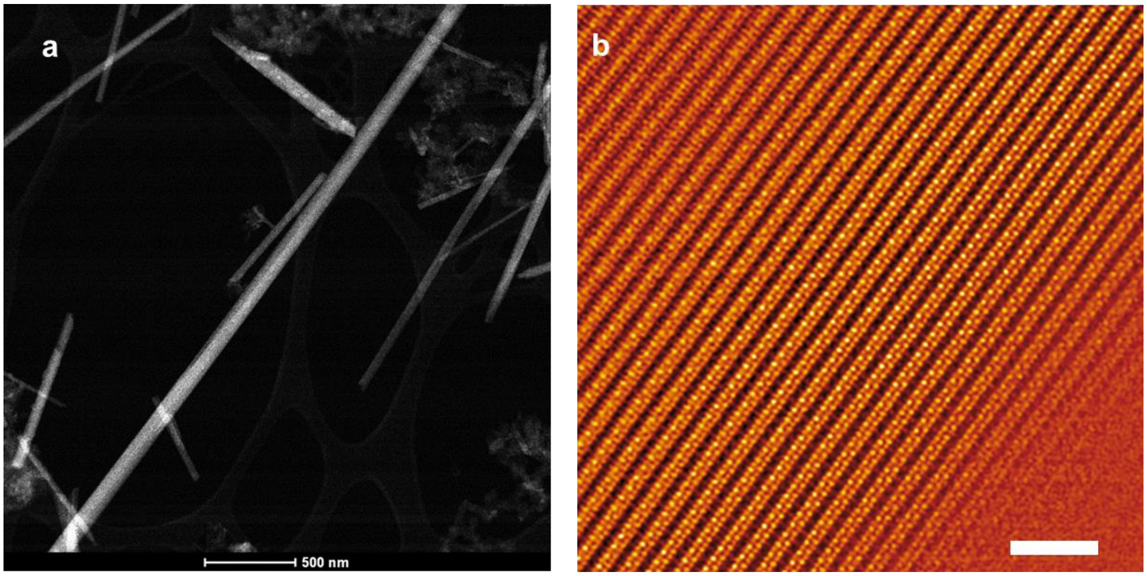

Supplementary Figure 46. Morphology characterization. (a) Low magnification TEM and (b) high-resolution HAADF-STEM images of fully discharged MnO_2 cathode. Scale bar: 2 nm in (b).

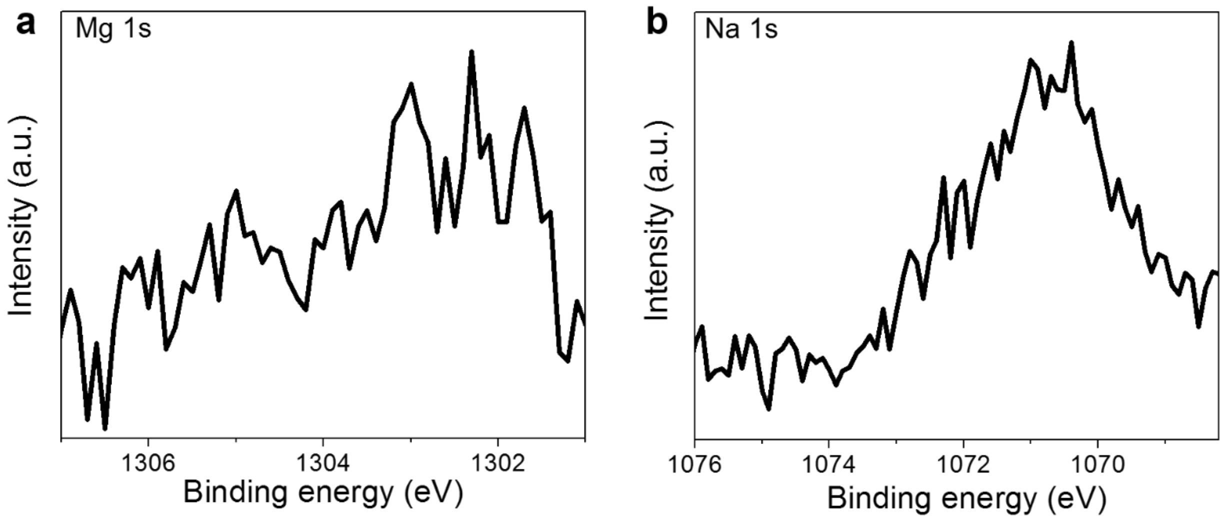

Supplementary Figure 47. XPS analysis. XPS spectra of (a) Mg 1s and (b) Na 1s of Zn-Mn alloy anode in Mg and Na-containing seawater-based electrolytes after cycling.

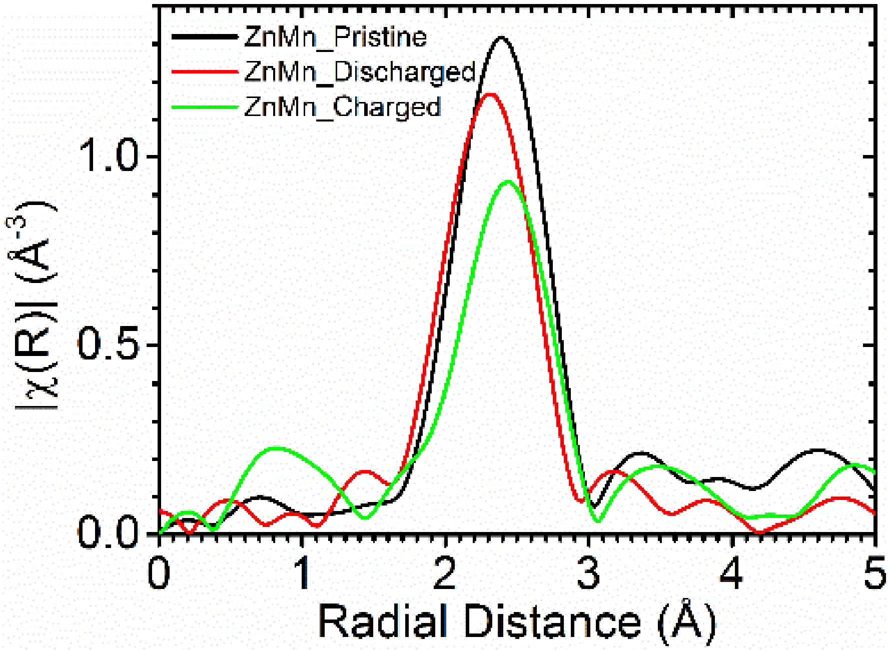

Supplementary Figure 48. Fourier transform of Mn K-edge EXAFS of Zn-Mn alloy at different cycling states (pristine, fully discharged (DC), and fully charged (C) Zn-Mn).

References

1. Quéré D. Wetting and Roughness. *Annual Review of Materials Research* **38**, 71-99 (2008).

2. Cebeci FÇ, Wu Z, Zhai L, Cohen RE, Rubner MF. Nanoporosity-Driven Superhydrophilicity: A Means to Create Multifunctional Antifogging Coatings. *Langmuir* **22**, 2856-2862 (2006).

3. Wang P, Zhang D, Qiu R, Hou B. Super-hydrophobic film prepared on zinc as corrosion barrier. *Corrosion Science* **53**, 2080-2086 (2011).

4. Tang H, *et al.* Synthesis and properties of hydroxyapatite-containing coating on AZ31 magnesium alloy by micro-arc oxidation. *Applied Surface Science* **400**, 391-404 (2017).

5. Yin YB, *et al.* Dendrite-Free Zinc Deposition Induced by Tin-Modified Multifunctional 3D Host for Stable Zinc-Based Flow Battery. *Adv Mater* **32**, (2020).

6. Parker JF, Chervin CN, Nelson ES, Rolison DR, Long JW. Wiring zinc in three dimensions re-writes battery performance-dendrite-free cycling. *Energ Environ Sci* **7**, 1117-1124 (2014).

7. Chao DL, *et al.* An Electrolytic Zn-MnO_2 Battery for High-Voltage and Scalable Energy Storage. *Angew Chem Int Edit* **58**, 7823-7828 (2019).

8. Zhao ZM, *et al.* Long-life and deeply rechargeable aqueous Zn anodes enabled by a multifunctional brightener-inspired interphase. *Energ Environ Sci* **12**, 1938-1949 (2019).

9. Shen C, *et al.* Graphene-Boosted, High-Performance Aqueous Zn-Ion Battery. *ACS Appl Mater Inter* **10**, 25446-25453 (2018).

10. Wang F, *et al.* Highly reversible zinc metal anode for aqueous batteries. *Nat Mater* **17**, 543-549 (2018).

11. Wang ZQ, *et al.* A MOF-based single-ion Zn^{2+} solid electrolyte leading to dendrite-free rechargeable Zn batteries. *Nano Energy* **56**, 92-99 (2019).

12. Zeng YX, *et al.* Dendrite-Free Zinc Deposition Induced by Multifunctional CNT Frameworks for Stable Flexible Zn-Ion Batteries. *Adv Mater* **31**, 1903675 (2019).

13. Zhao J, *et al.* High-performance flexible quasi-solid-state zinc-ion batteries with layer-expanded vanadium oxide cathode and zinc/stainless steel mesh composite anode. *Nano Energy* **62**, 94-102 (2019).

14. Wang Z, *et al.* A Metal-Organic Framework Host for Highly Reversible Dendrite-free Zinc Metal Anodes. *Joule* **3**, 1289-1300 (2019).

15. Deng CB, *et al.* A Sieve-Functional and Uniform-Porous Kaolin Layer toward Stable Zinc Metal Anode. *Adv Funct Mater* **30**, 2000599 (2020).

16. Zhao KN, *et al.* Ultrathin Surface Coating Enables Stabilized Zinc Metal Anode. *Adv Mater Interfaces* **5**, 1800848 (2018).

17. Yang Q, *et al.* Hydrogen-Substituted Graphdiyne Ion Tunnels Directing Concentration Redistribution for Commercial-Grade Dendrite-Free Zinc Anodes. *Adv Mater* **32**, 2001755 (2020).

18. Hao JN, *et al.* Designing Dendrite-Free Zinc Anodes for Advanced Aqueous Zinc Batteries. *Adv Funct Mater* **30**, 2001263 (2020).

19. Kang LT, *et al.* Nanoporous $CaCO_3$ Coatings Enabled Uniform Zn Stripping/Plating for Long-Life Zinc Rechargeable Aqueous Batteries. *Adv Energy Mater* **8**, 1801090 (2018).

20. Dong W, Shi JL, Wang TS, Yin YX, Wang CR, Guo YG. 3D zinc@carbon fiber composite framework anode for aqueous Zn-MnO_2 batteries. *RSC Adv* **8**, 19157-19163 (2018).

21. Kang Z, *et al.* 3D Porous Copper Skeleton Supported Zinc Anode toward High Capacity and Long Cycle Life Zinc Ion Batteries. *ACS Sustain Chem Eng* **7**, 3364-3371 (2019).

22. Tian Y, *et al.* Flexible and Free-Standing $Ti_3C_2T_x$ MXene@Zn Paper for Dendrite-Free Aqueous Zinc Metal Batteries and Nonaqueous Lithium Metal Batteries. *ACS Nano* **13**, 11676-11685 (2019).

23. Li CP, *et al.* Spatially homogeneous copper foam as surface dendrite-free host for zinc metal anode. *Chem Eng J* **379**, 122248 (2020).

24. Liang PC, *et al.* Highly Reversible Zn Anode Enabled by Controllable Formation of Nucleation Sites for Zn-Based Batteries. *Adv Funct Mater* **30**, 1908528 (2020).

25. Lee BS, *et al.* Dendrite Suppression Membranes for Rechargeable Zinc Batteries. *ACS Appl Mater Inter* **10**, 38928-38935 (2018).

26. Li M, *et al.* A Novel Dendrite-Free Mn^{2+}/Zn^{2+} Hybrid Battery with 2.3 V Voltage Window and 11000-Cycle Lifespan. *Adv Energy Mater* **9**, 1901469 (2019).

27. Zhang Q, *et al.* The Three-Dimensional Dendrite-Free Zinc Anode on a Copper Mesh with a Zinc-Oriented Polyacrylamide Electrolyte Additive. *Angew Chem Int Edit* **58**, 15841-15847 (2019).

28. Li W, Wang KL, Zhou M, Zhan HC, Cheng SJ, Jiang K. Advanced Low-Cost, High-Voltage, Long-Life Aqueous Hybrid Sodium/Zinc Batteries Enabled by a Dendrite-Free Zinc Anode and Concentrated Electrolyte. *ACS Appl Mater Inter* **10**, 22059-22066 (2018).

29. Wang LP, Li NW, Wang TS, Yin YX, Guo YG, Wang CR. Conductive graphite fiber as a stable host for zinc metal anodes. *Electrochim Acta* **244**, 172-177 (2017).

30. Wang SB, *et al.* Lamella-nanostructured eutectic zinc-aluminum alloys as reversible and dendrite-free anodes for aqueous rechargeable batteries. *Nat Commun* **11**, 1634 (2020).

31. Xia AL, Pu XM, Tao YY, Liu HM, Wang YG. Graphene oxide spontaneous reduction and self-assembly on the zinc metal surface enabling a dendrite-free anode for long-life zinc rechargeable aqueous batteries. *Appl Surf Sci* **481**, 852-859 (2019).

32. Shi XD, *et al.* Homogeneous Deposition of Zinc on Three-Dimensional Porous Copper Foam as a Superior Zinc Metal Anode. *ACS Sustain Chem Eng* **7**, 17737-17746 (2019).

33. Liu PG, Liu WF, Huang YP, Li PL, Yan J, Liu KY. Mesoporous hollow carbon spheres boosted, integrated high performance aqueous Zn-Ion energy storage. *Energy Storage Materials* **25**, 858-865 (2020).

34. Liu MQ, *et al.* Artificial Solid-Electrolyte Interface Facilitating Dendrite-Free Zinc Metal Anodes via Nanowetting Effect. *ACS Appl Mater Inter* **11**, 32046-32051 (2019).

35. Chao DL, *et al.* A High-Rate and Stable Quasi-Solid-State Zinc-Ion Battery with Novel 2D Layered Zinc Orthovanadate Array. *Adv Mater* **30**, 1803181 (2018).

36. Qiu HY, *et al.* Zinc anode-compatible in-situ solid electrolyte interphase via cation solvation modulation. *Nat Commun* **10**, 5374 (2019).

37. Zhao JW, *et al.* "Water-in-deep eutectic solvent" electrolytes enable zinc metal anodes for rechargeable aqueous batteries. *Nano Energy* **57**, 625-634 (2019).

38. Xu WN, *et al.* Diethyl ether as self-healing electrolyte additive enabled long-life rechargeable aqueous zinc ion batteries. *Nano Energy* **62**, 275-281 (2019).

39. Zhang C, *et al.* A $ZnCl_2$ water-in-salt electrolyte for a reversible Zn metal anode. *Chem Commun* **54**, 14097-14099 (2018).

40. Wang LJ, *et al.* A $Zn(ClO_4)_2$ Electrolyte Enabling Long-Life Zinc Metal Electrodes for Rechargeable Aqueous Zinc Batteries. *ACS Appl Mater Inter* **11**, 42000-42005 (2019).

41. Han Q, *et al.* An inorganic salt reinforced Zn^{2+}-conducting solid-state electrolyte for ultra-stable Zn metal batteries. *J Mater Chem A* **7**, 22287-22295 (2019).

42. Huang JQ, *et al.* Thickening and Homogenizing Aqueous Electrolyte towards Highly Efficient and Stable Zn Metal Batteries. *J Electrochem Soc* **166**, A1211-A1216 (2019).

43. Naveed A, Yang HJ, Yang J, Nuli YN, Wang JL. Highly Reversible and Rechargeable Safe Zn Batteries Based on a Triethyl Phosphate Electrolyte. *Angew Chem Int Edit* **58**, 2760-2764 (2019).

44. Zhang N, *et al.* Cation-Deficient Spinel $ZnMn_2O_4$ Cathode in $Zn(CF_3SO_3)_2$ Electrolyte for Rechargeable Aqueous Zn-Ion Battery. *J Am Chem Soc* **138**, 12894-12901 (2016).

45. Zhang N, *et al.* Rechargeable aqueous zinc-manganese dioxide batteries with high energy and power densities. *Nat Commun* **8**, 405 (2017).
