# Supplementary material for: Stable, high-performance, dendrite-free, seawater-based aqueous batteries
Source: Nat Commun. 2021 Jan 11;12:237. doi: 10.1038/s41467-020-20334-6 (PMC7801520; doi:10.1038/s41467-020-20334-6)
Supplement: Supplementary file 2 — Description of Additional Supplementary Files [file 41467_2020_20334_MOESM2_ESM.pdf]

Descriptions for Additional Supplementary files

Supplementary Movie 1. 3D COMSOL simulation of Zn plating for 50s.

Supplementary Movie 2. Dendrite growth on the pristine Zn surface.

Supplementary Movie 3. Differential optical video.

Supplementary Movie 4. Long time plating process (the color is plotted according to pixel intensity).

Supplementary Movie 5. Zn plating for 160s under a constant current of 80 mA cm^{-2}.

Supplementary Movie 6. Zn plating for 160s under a constant current of 80 mA cm^{-2} observed at a higher magnification.

Supplementary Movie 7. Zn stripping for 240s under a constant current of 80 mA cm^{-2}.

Supplementary Movie 8. Zn stripping for 240s under a constant current of 80 mA cm^{-2} observed at a higher magnification.

Supplementary Movie 9. Zn plating process during 0~1000s under a constant current of 80 mA cm^{-2}.

Supplementary Movie 10. Zn plating process during 1000~2800s under a constant current of 80 mA cm^{-2}.

Supplementary Movie 11. Zn plating process during 2800~4600s under a constant current of 80 mA cm^{-2}.

Supplementary Movie 12. Zn plating process during 4600~6400s under a constant current of 80 mA cm^{-2}.

Supplementary Movie 13. Zn plating process during 6400~8200s under a constant current of 80 mA cm^{-2}.

Supplementary Movie 14. COMSOL simulation of Zn plating for 30s.

Supplementary Movie 15. Zn plating on the 3D Zn-Mn alloy surface for 300s under a constant current of 80 mA cm^{-2} with the 2M $ZnSO_4$ in seawater as the electrolyte.

Supplementary Movie 16. Zn plating on the 3D Zn-Mn alloy surface for 300s under a constant

current of 80 mA cm^{-2} with the 2M $ZnSO_4$ and 0.1M $MnSO_4$ in seawater as the electrolyte.

Supplementary Movie 17. Zn plating on the 3D Zn-Mn alloy surface for 300s under a constant current of 80 mA cm^{-2} with the 2M $ZnSO_4$ and 0.1M $MnSO_4$ in DI water as the electrolyte.

Supplementary Movie 18. Zn plating on the pristine Zn surface 120s under a constant current of 80 mA cm^{-2} with the 2M $ZnSO_4$ and 0.1M $MnSO_4$ in seawater as the electrolyte.

Supplementary Movie 19. Zn plating on the 3D Zn-Mn alloy surface for 120s under a constant current of 80 mA cm^{-2} with the 2M $ZnSO_4$ and 0.1M $MnSO_4$ in seawater as the electrolyte.

Supplementary Movie 20. Zn plating on the plane Zn-Mn alloy for 900s under a constant current of 80 mA cm^{-2}.

Supplementary Movie 21. An electric fan powered by the flexible tandem ZABs.
